# An orthoflavivirus inhibitor targeting multifunctional NS2A protein, a previously unidentified target

**DOI:** 10.1371/journal.ppat.1014190

**Published:** 2026-05-05

**Authors:** Doortje Borrenberghs, Kitti Wing Ki Chan, Sven Van Brandt, Steffen Jaensch, Milly Ming Ju Choy, Peggy Geluykens, Asmae El Bouayadi, Dax Lauwers, Pradeep Bist, Bart Stoops, Jeroen Van de Ven, Peter Vermeulen, Sarah Megens, Kim Thys, Daniëlle Peeters, Suzanne J. F. Kaptein, Johan Neyts, Subhash G. Vasudevan, Anil Koul, Olivia Goethals

**Affiliations:** 1 Communicable diseases, Johnson & Johnson Pharmaceutical Research and Development, Beerse, Belgium; 2 Center of Global Health Discovery - Flavivirus, Program in Emerging Infectious Diseases, Duke-NUS Medical School, Singapore, Singapore; 3 Janssen Research and development, Johnson & Johnson Pharmaceutical Research and Development, Beerse, Belgium; 4 Discovery, Charles River Laboratories International Inc, Beerse, Belgium; 5 KU Leuven Department of Microbiology, Immunology and Transplantation, Rega Institute for Medical Research, Virology, Antiviral Drug & Vaccine Research Group, Leuven, Belgium; 6 Institute of Biomedicine and Glycomics, Griffith University, Queensland, Australia; SUNY Upstate Medical University: State University of New York Upstate Medical University, UNITED STATES OF AMERICA

## Abstract

Orthoflaviviruses, such as dengue virus (DENV), Zika virus (ZIKV), West Nile virus (WNV), Japanese encephalitis virus (JEV), and yellow fever virus (YFV), constitute a significant public health concern with billions of people at risk of infection. Climate change and the expanding geographical distribution of mosquito vectors transmitting orthoflaviviruses have increased their potential to cause large-scale disease outbreaks. The frequency and severity of disease outbreaks highlight the urgent need for a broad-spectrum antiviral agent targeting orthoflaviviruses. In this work, we conducted a comprehensive morphological profiling of approximately 200,000 small molecules through a fluorescence-based high-content imaging platform, which led to the identification of a singular small molecule exhibiting broad-spectrum activity against orthoflaviviruses. Subsequent hit deconvolution against DENV serotype 2 (DENV-2) revealed NS2A protein as a novel therapeutic target. Mechanistically, JNJ-1953 inhibits viral RNA synthesis, as demonstrated by robust reductions in intracellular viral RNA and infectious virus production. Additional experiments show that JNJ-1953 further impacts viral RNA packaging and interferes with the interaction between NS2A and prM, rendering the molecule a multimodal inhibitor.

## Author summary

Orthoflaviviruses, including Dengue, West Nile and ZIKV, cause significant global disease yet lack broadly effective antiviral drugs. To address this unmet need, we used a cell-based high-content screening approach to discover a novel class of small molecules that inhibit a previously underexplored viral protein, NS2A. Guided by structure-activity relationship optimization, we advanced a lead compound, JNJ-1953, with chemical- and virus-dependent activity against multiple orthoflaviviruses. Mechanistic studies revealed that this compound engage NS2A protein and act through complementary antiviral mechanisms: it reduces viral RNA synthesis, impairs packaging of viral genomes into new virus particles, and disrupts the interaction between NS2A and the viral prM protein. Together, these complementary mechanisms block key steps in the orthoflavivirus replication cycle. This work is the first to report a series of small molecules with activity against different orthoflaviviruses that target NS2A protein, establishing NS2A protein as a promising antiviral target. Ultimately, targeting NS2A protein could enable new therapeutic strategies to reduce the burden of orthoflavivirus infections worldwide.

## 1. Introduction

Orthoflaviviruses, such as dengue virus (DENV), Zika virus (ZIKV), West Nile (WNV), yellow fever (YFV), and Japanese encephalitis virus (JEV) are mosquito-borne human pathogens known for causing a wide range of diseases in humans, including dengue fever, yellow fever, and viral encephalitis. Collectively, they threaten more than half of the global population and gained increasing attention in recent years due to their ability to cause significant outbreaks and global health concerns, especially in areas that are naïve to infection [1,2].

The development of vaccines and therapeutics against orthoflaviviruses has proven challenging. While licensed vaccines are available for certain mosquito-borne orthoflaviviruses, including DENV, YFV and JEV, the vaccine development process is complicated by several factors. These include suboptimal safety profiles, the need to establish a durable protective immune response, and the occurrence of cross-reactive antibodies, which can lead to antibody-dependent enhancement of disease [3,4]. The COVID-19 pandemic also revealed major points of concern regarding distribution and availability of vaccines to low-income countries. In addition, the pandemic highlighted the importance of developing drugs for antiviral treatment and/or prophylaxis. Currently, no antiviral is clinically available for prevention and treatment of any of these orthoflavivirus infections.

Orthoflavivirus virions are 40–60 nm in diameter structures that contain a single-stranded plus-sense RNA genome (~11 kb), which is translated into a single polyprotein precursor, that is co- and post-translationally cleaved into three structural proteins (capsid [C], pre-membrane [prM] and envelope [E]) and seven non-structural proteins (NS1, NS2A, NS2B, NS3, NS4A, NS4B and NS5) by host and viral (NS2B-NS3) proteases [5,6]. The non-structural proteins form the replication complex which is associated with the endoplasmic reticulum (ER) membrane [7–9]. Some of the NS proteins drive the viral replication process, such as NS1 protein [10,11], NS3 helicase [12], NS2B-NS3 protease [12–14], NS5 methyl-transferase [15–18] and NS5 RNA-dependent RNA polymerase [19,20]. Other NS proteins (NS2A, NS4A and NS4B) have no enzymatic activity ascribed but contain transmembrane domains associated with the ER membrane, serving as scaffolds for the replication complex [8,21–23]. Apart from serving as a membrane anchoring point and being a cofactor in the formation of the viral replication complex, the NS4A protein also interacts with host factors involved in immune responses, and plays a role in viral pathogenesis, inducing cellular stress responses and apoptosis [24]. Several important functions have been ascribed to NS4B protein [25]. Like the NS4A protein, the NS4B protein plays a crucial role in the formation and organization of the viral replication complexes by aiding in the remodeling of the host cell membrane to create vesicle packets. NS4B protein can modulate host immune responses and has been implicated in viral pathogenesis. More recently, *in vitro* studies revealed that NS4B protein dissociates NS3 protein from single-stranded RNA and enhances NS3 helicase activity [26,27]. NS2A protein plays a crucial role in various stages of the viral replication cycle. The protein appears to be a central hub in the virion packaging process, by coordination of viral and host factors, host antiviral response and recruitment of the viral RNA [2,5,7,21,28–40]. NS2A protein has a documented role in viral replication, by interacting with the 3’ untranslated region (3’UTR) of the viral RNA as well as with other viral components involved in virion assembly to coordinate genome encapsulation and assembly [7,39,40]. Furthermore, NS2A protein is also suggested to function as a viroporin, because of its oligomerization, potentially helping to direct nascent viral RNAs to the sites of virion assembly [41]. One single virion consists of multiple copies of prM and E, therefore each NS2A molecule may aid in the coordination of the complex virion assembly process [42].

Recent advances in high-content imaging (HCI), an image-based technology in high throughput format, allow both visualization of the virus and changes in cellular phenotypes induced by compound treatment [43,44]. This approach has been used in recent antiviral screening campaigns and has been successful in identifying inhibitors against various viruses, such as Hepatitis B virus [45], coronavirus [46,47], and ZIKV [48].

Here, a cellular-based HCI screening campaign resulted in the identification of a novel, multimodal orthoflavivirus inhibitor. Through structure-activity relationship (SAR) optimization, a lead compound was identified with chemical- and virus-dependent activity observed across orthoflaviviruses. Target engagement identified NS2A protein as a previously unidentified target, affected by the inhibitor through complementary mechanisms.

## 2. Results

### 2.1. High-content imaging (HCI) antiviral assay against DENV-2

**Fig 1:**
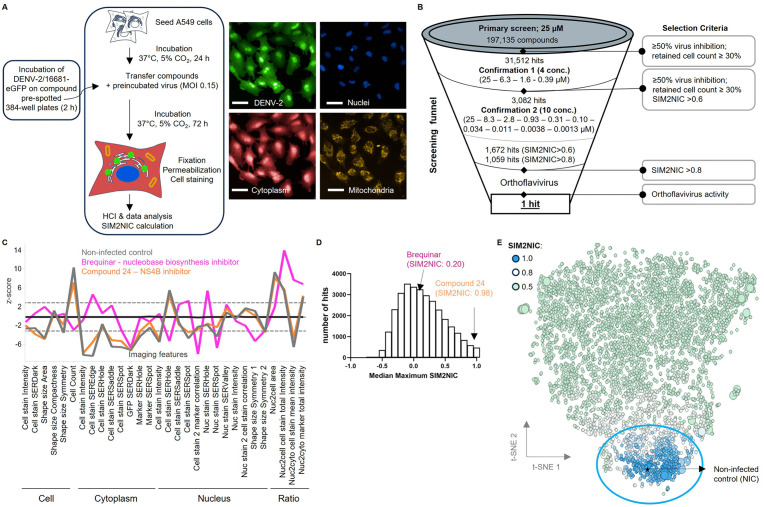
High-content imaging antiviral assay based on DENV-2/16681-GFP in A549 cells. **A** Experimental set up of the used HCI fluorescence platform. Representative images of DENV-2/16681-GFP infective A549 cells. Images from CV7000 Yokagawa showing eGFP signal coming from the DENV-2/16681-GFP infection (eGFP, green), Hoechst staining (nuclei, blue), MitoTracker orange staining (mitochondria, Orange) and Cell-Mask Deep red staining (cytoplasm and nucleus, Red). Scale bar: 50 µM. **B** High level flow of the screening funnel from the primary screen to the identification of hits with orthoflavivirus broad-spectrum potential. **C** Morphological profiles based on 30 features for each compound, here shown for two reference compounds, Brequinar [49] (a selective inhibitor of the enzyme dihydroorotate dehydrogenase) and compound 24 [50] (a DENV NS4B inhibitor). **D** Results of the hit deprioritization using SIM2NIC. The maximum SIM2NIC score over all concentration at which a compound achieved ≥ 50% virus inhibition and retained cell count ≥ 30% (relative to infected control (0-line) was computed and plotted. Non-infected control cells had a SIM2NIC value of 0.99 (median of individual control wells). The SIM2NIC values obtained for the reference compounds Brequinar and compound 24 were 0.20 and 0.98, respectively. **E** T-SNE clustering of full feature analysis of hits from the confirmation screen identifies clusters of compounds with similar phenotypes. The phenotypic cluster of hits with a SIM2NIC > 0.8 closely correlates with the non-infected control (NIC; star symbol). The reference compounds used to select the 30 features needed for calculation of the SIM2NIC value are presented by large circles. Every small circle, represent a test compound.

In a quest for novel orthoflavivirus inhibitors, a fluorescence-based HCI platform in A549 cells was set up (Figs 1A and S1A) using DENV-2 as a representative orthoflavivirus. Using this optimized and validated platform (S1B-S1C Fig), we screened approximately 200,000 compounds at a single concentration of 25 µM, originating from the Johnson & Johnson library and which were selected based on their biological and chemical diversity (Fig 1B). In addition to viral infection (eGFP expression) and cell viability (cell count normalized to infected cells), image-based morphological profiling of cells was carried out during a first confirmation run. Morphological profiles were evaluated based on 30 cellular features determined during screen optimization, including intensity, texture, and shape properties (Fig 1C) [43,45–48]. The morphological profile induced by each compound in infected cells was compared to that of non-infected controls on the same plate and a “similarity to non-infected control” (SIM2NIC) score was calculated (Fig 1D and 1E). A SIM2NIC score close to 1 reflects a high correlation in morphology to non-infected control, and direct-acting antivirals are expected to have high SIM2NIC values close to 1. Two examples are shown: one nucleobase biosynthesis inhibitor, Brequinar [49], and a specific DENV NS4B inhibitor, compound 24 [50]. While compound 24 exhibits a morphological profile similar to that of the non-infected control, with a SIM2NIC close to 1 (SIM2NIC: 0.98), the morphological profile of Brequinar diverges significantly, resulting in a markedly low SIM2NIC (SIM2NIC: 0.20) (Fig 1C and 1D). Compounds blocking virus infection by more than 50%, exhibiting a decrease in cell count of less than 70% compared to infected control cells and having a SIM2NIC > 0.6, were selected for further confirmation screening (Fig 1B). The hit rate from the primary screening after confirmation in 4 concentrations was 1.5%, resulting in 3,082 hits, which were next tested at 10 concentrations (Fig 1B). A total of 1,672 hits exhibited an antiviral activity of > 50%, a cell count > 30% and a SIM2NIC > 0.6. From this pool, 1,059 hits with a SIM2NIC threshold higher than 0.8 were further evaluated (Fig 1B). Frequent hitters (i.e., compounds frequently identified as “hits” across previous internal high-throughput screening (HTS) campaigns) were eliminated. Next, compounds were clustered by chemical similarity and ranked based on their overall antiviral DENV-2 potency within each cluster. For each cluster the available SAR is evaluated, noting whether hits were singletons or accompanied by close analogues that indicate a dynamic SAR. Chemical attractiveness was assessed by predicted promiscuity (low), favorable physiochemical properties (lipophilicity and molecular weight), absence of pan-assay interference compounds (PAINS) [51], and lack of reactive or undesirable functional groups. The resulting hit classes of interest were further explored for their potential orthoflavivirus activity against DENV-1/-3/-4, ZIKV, WNV, JEV and YFV (Fig 1B).

### 2.2. Identified hit JNJ-3644 shows activity across orthoflaviviruses

**Fig 2:**
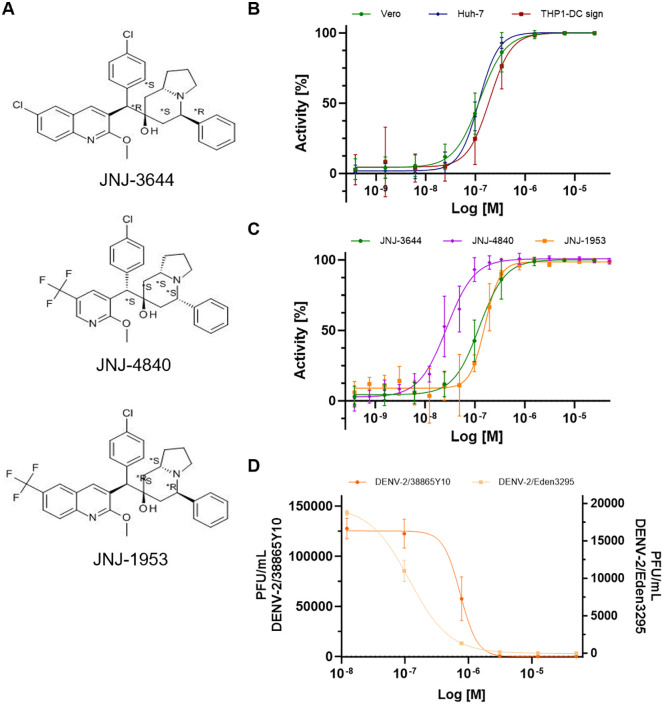
Orthoflavivirus antiviral activity evaluation of the identified hit series. **A** Structure of compound JNJ-3644, JNJ-1953 and JNJ-4840. **B** Antiviral activity (% Inhibition eGFP expression) of JNJ-3644 against DENV-2/16681-eGFP in Vero cells, Huh7 hepatoma cells and human monocytic leukemia THP1 cells expressing the DC-SIGN receptor. **C** Antiviral activity (% Inhibition eGFP expression) of JNJ-3644, JNJ-1953 and JNJ-4840 against DENV-2/16681 in Vero cells. **D** Antiviral activity (plaque forming units per mL) of JNJ-1953 against DENV-2/Eden3295 (right Y-axis) and DENV-2/38865Y10 (left y-axis) in Huh7 cells. Data represent mean values ± SD from at least two independent experiments.

One hit, 7-[(6-chloro-2-methoxy-3-quinolyl)-(4-chlorophenyl)methyl]-5-phenyl-2,3,5,6,8,8a-hexahydro-1H-indolizin-7-ol (JNJ-3644; Fig. 2A Top), exhibited nanomolar (nM) antiviral potency against DENV-2/16681-eGFP in various cell lines (Vero, Huh7 and THP1-DC sign), accompanied with high selectivity (SI, selective index) (Table 1 and Fig 2B). JNJ-3644 has 4 chiral centers, resulting in 8 different enantiomers. The hit compound had an EC_50_ value of 0.18 µM with a SI of 72, while the other enantiomers had EC_50_ values ranging from 0.85 to 4.6 µM, with selective indices between 4 and 7 (S1 Table and Fig 2A). Next, JNJ-3644 was tested in a tetravalent dengue antiviral assay using RT-qPCR as readout, and the EC_50_ for the DENV serotypes ranged from 0.089 to 1.0 µM (Table 1 and S2B Fig). Initial testing of JNJ-3644 (4 concentrations) in different orthoflavivirus assays showed EC_50_ values of around 0.77 µM (ZIKV, JEV), 1.96 µM (YFV) and 2.65 µM (WNV).

**Table 1:** Antiviral activity of JNJ-3644 against a set of different orthoflaviviruses.

Cells	Virus	JNJ-3644			
		EC_50_ [µM]	EC_90_ [µM]	CC_50_ [µM]	SI
Vero	DENV-2/16681	0.12 ± 0.075	0.45 ± 0.47	12 ± 5.9	100
Huh7	DENV-2/16681	0.11 ± 0.023	0.31 ± 0.074	4.7 ± 1.4	43
THP1-DC sign	DENV-2/16681	0.1 ± 0.080	0.58 ± 0.27	7.8 ± 4.5	41
Vero	DENV-1/TC974 666	0.32 ± 0.17	2.4 ± 0.30	6.6 ± 1.4	21
Vero-GFP	DENV-1/TC974 666	0.68 ± 0.34	3.9 ± 2.2	11	
Vero	DENV-2/16681	0.090 ± 0.020	0.6 ± 00.70	7.9 ± 3.2	88
Vero-GFP	DENV-2/16681	0.099 ± 0.14	0.90 ± 0.95	11	
Vero	DENV-3/H87	0.59 ± 0.25	4.6 ± 0.89	4.7 ± 0.40	8
Vero-GFP	DENV-3/H87	0.54 ± 0.52	3.5 ± 4.3	9	
Vero	DENV-4/H241	1.0 ± 0.60	9.6 ± 8.2	10 ± 9.6	10
Vero-GFP	DENV-4/H241	0.46 ± 0.59	2.7 ± 3.0	10	
Vero	WNV/NY-99	2.7	ND	9.7	4
Vero-GFP	WNV/B956	0.39 ± 0.31	1.6 ± 1.8	12 ± 6.4	31
Vero	ZIKV/MR766	0.77	ND	8.2	11
Vero-GFP	ZIKV/MP1751	2.0 ± 1.2	4.8 ± 2.7	15 ± 7.1	8
Huh7	YFV/17D	1.9	ND	20	10
Vero-GFP	YFV/17D	1.2 ± 0.59	4.5 ± 5.6	12 ± 5.2	10
Vero	JEV/SA14-14-2	0.77	ND	9.7	13

The initial antiviral data of JNJ-3644 for WNV/NY-99, ZIKV/MR766, YFV/17D and JEV/SA14-14-2 only represent one test experiment, subsequent testing of the compound against the orthoflaviviruses was performed in the Vero-GFP antiviral assay where mean values from at least two independent experiments are shown. EC_50_: 50% effective concentration. EC_90_: 90% effective concentration; CC_50_: 50% cytotoxic concentration; Selectivity index (SI): ratio CC_50_/EC_50_. ND: Not determined.

No marked antiviral activity was detected against a selection of other RNA and DNA viruses (S2 Table). More than 300 compounds based on the same chemical series as JNJ-3644 were synthesized and tested in different orthoflavivirus antiviral assays. Compound efficacy against some of the orthoflaviviruses varied. For instance, compared to JNJ-3644, JNJ-4840 (Fig 2A Middle) had a 4–5-fold better activity against DENV-2 (Fig 2C and Table 2) and YFV but with a 3-fold lower efficacy against DENV-3 (S2C Fig). Another compound, JNJ-1953 (Fig 2A Bottom), had similar antiviral potency (Table 2 and Figs 2C and S2C) but improved microsomal clearance and mitochondrial toxicity compared to JNJ-3644 (Table 3). For this reason, most of the further experiments were executed using JNJ-1953 as the lead compound. Like the efficacy against DENV-2/16681, JNJ-1953 retained nM potency against DENV-2 clinical isolates DENV-2/EDEN3295 and DENV-2/38865Y10 (Table 2 and Fig 2D) and ZIKV (S2D Fig). Since there is increased risk of severe dengue arising from secondary heterotypic infection in humans, a phenomenon known as antibody-dependent enhancement (ADE), we next evaluated the efficacy of JNJ-1953 in an in vitro monocytic cell line THP-1 ADE infection model [52]. Analogous to the efficacy observed in direct infection on different mammalian cell lines, JNJ-1953 exhibited low nM potency (EC_50_ = 50 nM; Table 2) against ADE infection. Collectively the nM potency of JNJ-1953 in both direct and ADE infections supports further development of this compound as a potential antiviral against orthoflaviviruses.

**Table 2:** Antiviral activity of JNJ-4840 and JNJ-1953 against different orthoflaviviruses.

Cells	Virus	JNJ-4840				JNJ-1953			
		EC_50_ [µM]	EC_90_ [µM]	CC_50_ [µM]	SI	EC_50_ [µM]	EC_90_ [µM]	CC_50_ [µM]	SI
Vero	DENV-2/16681	0.027 ± 0.011	0. 11 ± 0.063	5.1 ± 0.91	189	0.16 ± 0.043	0.34 ± 0.065	12 ± 11	75
Vero-GFP	DENV-1/TC974 666	0.56 ± 0.098	3.2 ± 1.9	7.1 ± 0.73	13	0.26 ± 0.03812	0.88 ± 0.026	5.4 ± 0.25	21
Vero-GFP	DENV-2/16681	0.041 ± 0.018	0.14 ± 0.041		173	0.14 ± 0.025	0.50 ± 0.18		39
Vero-GFP	DENV-3/H87	0.87 ± 0.76	5.9 ± 3.3		8	0.27 ± 0.18	0.75 ± 0.19		20
Vero-GFP	DENV-4/H241	0.70 ± 0.33	5.6 ± 2.8		10	0.36 ± 0.096	1.3 ± 0.36		15
Vero-GFP	WNV/B956	0.34 ± 0.039	0.44 ± 0.14	10 ± 0.12	29	0.2 ± 50.16	1.2 ± 0.46	9.0 ± 0.44	36
Vero-GFP	ZIKV/MP1751	1.8 ± 2.1	3.4 ± 1.7	15 ± 3.7	8	1.5 ± 0.6	1.8 ± 0.1	>25	17
Vero-GFP	YFV/17D	0.58 ± 0.084	4.4 ± 1.7	7.1 ± 0.26	12	2.2 ± 1.7	2.3 ± 0.06	13 ± 3.9	6
Huh7	DENV-2/EDEN3295	NT				0.29 ± 0.10	ND	61	288
Huh7	DENV-2/38865Y10	NT				0.27 ± 0.10	ND	61	178
THP1 ADE	DENV-2/38865Y10	NT				0.048 ± 0.0059	ND	ND	

Antiviral data represents mean values from at least two independently performed experiments. The toxicity of Huh7 infected with DENV-2/EDEN3295 and DENV-2/38865Y10 in presence of JNJ-1953 represents one experiment. EC_50_: 50% effective concentration. CC_50_: 50% cytotoxic concentration; EC_90_: 90% effective concentration; Selectivity index (SI): ratio CC_50_/EC_50_; NT: Not tested. ND: Not determined.

**Table 3:** ADME-Tox profile of compounds JNJ-3644, JNJ-1953 and JNJ-4840.

Parameter		JNJ-3644	JNJ-1953	JNJ-4840
CHI logD	pH 2.6	2.7	3.5	2.1
cLogP		6.7	6.9	5.6
Equilibrium solubility	pH 2 (µg/mL)	ND	94	ND
HTeqSol	pH 4 (µg/mL)	ND	100	ND
	pH 7.4 (µg/mL)	ND	62	ND
Intrinsic clearance cyprotex microsome	Human – Clint (µL/min/mg Prot)	39	<7.7	115
	Mouse – Clint (µL/min/mg Prot)	101	30	>347
Plasma protein binding		>99%	>99%	>99%
Cytotoxic screen in HepG2 cells	IC_20_ (µM)	11	11	15
Mitochondrial toxicity	(Glu/Gal) ratio	>100/18 (>5.7)	99/29 (3.4)	65/33 -2
CYP inhibition in human liver microsomes, Cytochrome P450 proteins family	1A2 – CEC (IC_50_ µM)	>20	>20	NT
	2C8 – DBF (IC_50_ µM)	>20	>20	NT
	2C9 – MFC (IC_50_ µM)	>20	>20	NT
	2C19 – CEC (IC_50_ µM)	>20	>20	NT
	2D6 – AMMC (IC_50_ µM)	15	12	NT
	3A4 – DBF (IC_50_ µM)	>20	>20	NT
Cardiovascular safety	Calcium		>30 µM	
	Sodium		>30 µM	
	Potassium channel (hERG)		>30 µM	

CHI LogD: Chromatographic hydrophobicity index; Clint: Intrinsic clearance; CYP: Cytochrome P450; hERG: human ether-à-go-go-related gene; NT: Not tested; ND: Not determined. Colors are provided for visual guidance only; color coding indicates relative preferability: green = preferred, orange = intermediate, red = not preferred.

### 2.3. JNJ-1953 acts on a viral replication step before virus secretion

**Fig 3:**
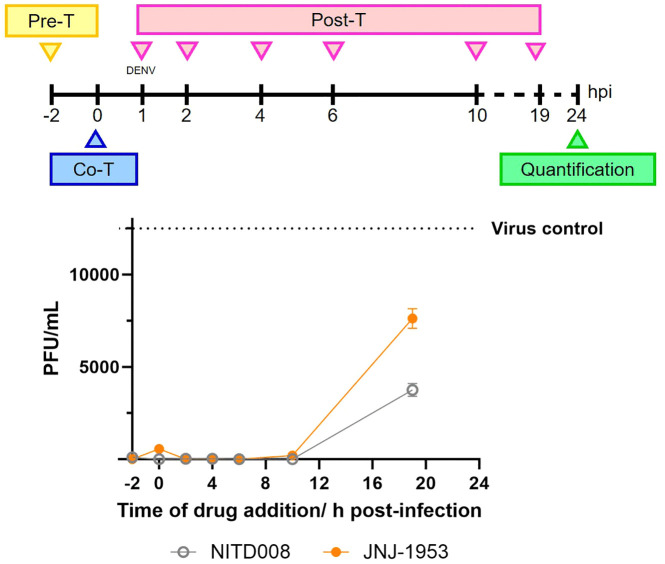
Time of drug addition studies of JNJ-1953. Huh7 cells were either treated with JNJ-1953 (orange symbol/line) 2 h prior to DENV-2/EDEN3295 infection (pre-treatment; Pre-T), infected with DENV-2/EDEN3295 in the presence of the compound (co-treatment; Co-T), or post-treated with the compound after 1 h DENV-2/EDEN3295 infection at the indicated hours post-infection (post-treatment; Post-T) as shown in the schematic. At 24 hours post-infection, plaque quantification of the culture supernatants was assessed. NITD008 was included as reference compound [53] (gray symbol/line). Data is presented as line graph showing the average PFU/mL with standard deviation of the treated samples compared to the untreated infected control (gray dotted line) obtained from two independent experiments with duplicates.

As a first step towards understanding the antiviral mechanism of JNJ-1953, a time-of-addition (TOA) experiment was performed to map the viral replication step(s) inhibited by the compound as depicted in the schematic (Fig 3; Top). The Fig 3 bottom panel shows the antiviral effect of JNJ-1953 added at different timings during infection. Pre-exposure of cells to 5 µM of JNJ-1953, 2 hours before infection (pre-treatment, Pre-T, -2 hours) or co-treatment (Co-Treatment, Co-T, 0 hours) during virus infection, resulted in > 90% virus inhibition, likely due to the compound’s high potency, similar to the well-characterized RNA virus polymerase inhibitor NITD008 [53]. Notably complete virus inhibition with respect to the mock treated virus control was observed when JNJ-1953 was added between 2–10 h post-infection (Post-Treatment, Post-T), a time window that corresponds to the release of viral RNA, protein translation and initiation of RNA synthesis [54]. Only a partial virus inhibition of ~40% was observed when the compound was added at 19 h post-infection, a timepoint when newly formed virions are thought to be secreted [54]. Collectively, these results suggest that JNJ-1953 exerts its antiviral activity in replication step(s) before virus secretion.

### 2.4. Resistance-associated mutations point NS2A as the target of JNJ-1953

**Fig 4:**
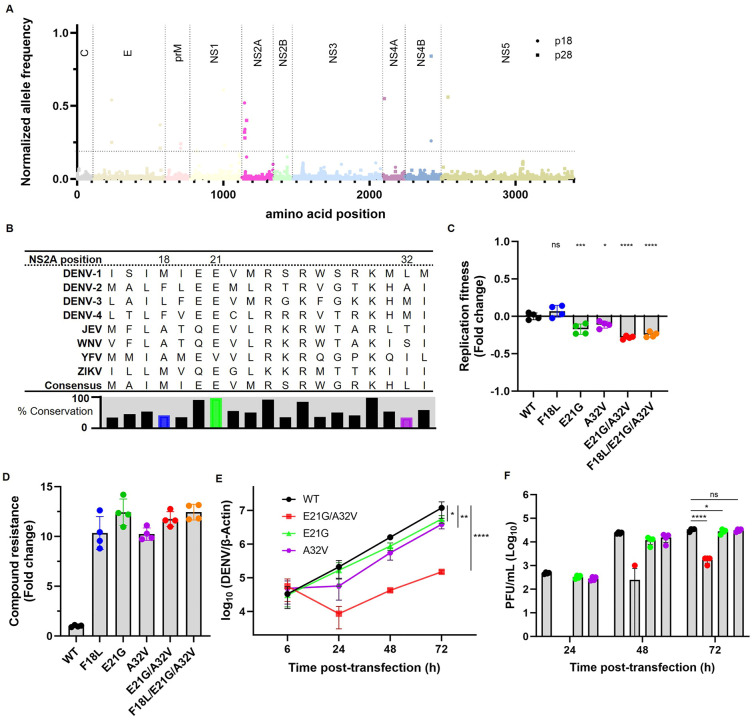
*In vitro* resistance selection experiments identify NS2A protein as target. **A** Allele frequency distributions of the polyprotein of two passaged (p18 and p28) DENV-2/16681 viruses which were exposed to increasing drug concentrations of JNJ-1953, highlighting mutations within NS2A protein (pink region). **B** Sequence alignment of a part of the NS2A proteins among DENV 4 serotypes, ZIKV, WNV, JEV, and YFV. The consensus and conservation percentage are indicated below each amino acid. **C** Effect of the single, double and triple NS2A resistance mutations (F18L, E21G, A32V, E21G/A32V and F18L/E21G/A32V) on the replication fitness of the subgenomic DENV-2/16681 reporter replicon. Data shown represents two independent experiments with two biological replicates each (total n = 4). Values were Log_2_-transformed and comparisons between WT and each mutant were performed by one-way ANOVA followed by Dunnett’s multiple comparisons test. **D** Level of compound resistance to JNJ-1953 imposed by NS2A resistance mutations (NS2A^F18L^, NS2A^E21G^, NS2A^A32V^, NS2A^E21G/A32V^ and NS2A^F18L/E21G/A32V^). **E** Intracellular viral RNA replication of WT and the NS2A mutant (NS2A^E21G^ (green), NS2A^A32V^ (purple) and NS2A^E21G/A32V^ (red)) DENV-2/EDEN3295 viruses determined by RT-qPCR. **F** Infectious-virus titers of WT and mutant DENV-2/EDEN3295 viruses (NS2A^E21G^ (green), NS2A^A32V^ (purple) and NS2A^E21G/A32V^ (red)) in the culture supernatants of electroporated cells as determined by standard BHK-21 plaque assay. Data presented in E and F are from two independent experiments with technical readings. The difference in the kinetics of the (E) intracellular viral RNA synthesis and (F) virus titers between WT and the various mutants are compared by two-way ANOVA with Geisser-Greenhouse correction and statistical significance are indicated (* - p < 0.05; ** - p < 0.01; *** - p < 0.001; **** - p < 0.0001).

To identify the molecular target of JNJ-3644 and JNJ-1953, drug-resistant DENV-2 variants were selected during *in vitro* resistance selection (IVRS) experiments and subsequently characterized by next-generation sequencing (NGS). Sequence alignment between the parental and drug-resistant DENV-2 variants (Fig 4A) of three independent IVRS efforts revealed three single-nucleotide amino acid substitutions in NS2A: F18L, E21G, and A32V, which were not present in the in-parallel-passaged untreated cultures and compared to the other drug-resistant substitutions were present in all drug-resistant variants (S3-S5 Tables). The three observed mutations (NS2A^F18L^, NS2A^E21G^ and NS2A^A32V^) are closely located near or within an N-terminal basic cluster (Residues 17–22; Fig 4B). Alignment of 17,328 NS2A protein sequences of the different orthoflaviviruses (DENV serotypes 1–4, JEV, WNV, YFV and ZIKV) collected from the Bacterial and Viral Bioinformatics Research Center (BV-BRC) database (Fig 4B) showed 96.8% (99.8% among DENV-2 sequences) conservation for glutamic acid (E) 21 (NS2A^E21^). Interestingly, no glycine (G) was observed in any of the orthoflavivirus NS2A protein sequences at amino acid position 21 (S6 Table). For the amino acid at position 32, 99.8% of the DENV-2 NS2A protein sequences had an alanine (A; NS2A^A32^) (Fig 4B), while 0.64% of the orthoflavivirus NS2A proteins contained a valine (V) at this position. For the amino acid at position 18 of the NS2A protein, 99.35% of the DENV-2 NS2A protein sequences had a phenylalanine (F; NS2A^F18^) at position 18 (S6 Table). However, the conservation of this F was remarkably lower (38%) among all orthoflaviviruses (Fig 4B), and a leucine (L) is observed in approximately 16% of the different orthoflavivirus NS2A protein sequences, and in 100% of the DENV-3 NS2A protein sequences (S6 Table). To assess the impact of these mutations on viral replication fitness and inhibitor resistance, the three mutations (NS2A^F18L^, NS2A^E21G^, and NS2A^A32V^) were individually or in combination (NS2A^E21G/A32V^ and NS2A^F18L/E21G/A32V^) introduced into a subgenomic DENV-2/16681 reporter replicon via site-directed mutagenesis (S3A Fig). The NS2A^F18L^ mutation alone did not affect replication, whereas the introduction of NS2A^E21G^ and NS2A^A32V^ or their combinations (NS2A^E21G/A32V^ and NS2A^F18L/E21G/A32V^) resulted in reduced replication efficiency (Figs 4C and S3B). The greater reduction in replicative fitness observed with the double and triple mutants suggests that mutations within the NS2A protein may act synergistically to impair virus replication. The antiviral activity of JNJ-1953 against these mutants was determined in a transient replicon assay. While JNJ-1953 efficiently inhibited wild-type (WT) DENV-2/16681 replication, all mutant viruses showed a 9–13-fold reduced susceptibility to JNJ-1953 (Table 4), suggesting that the mutations (NS2A^F18L^, NS2A^E21G^ and NS2A^A32V^) impact viral susceptibility. In addition, similar reduced susceptibility to compounds JNJ-3644 and JNJ-4840 were observed (S7 Table). Although the mutations resulted in reduced replicative fitness, this did not directly correlate with the level of resistance conferred by these mutations (Table 4 and Fig 4D).

**Table 4:** Antiviral activity of JNJ-1953 against WT and mutant DENV-2 subgenomic constructs and DENV-2/EDEN3295.

	pFK-sgDVs-R2A		DENV-2/EDEN3295	
Mutations	EC_50_ [µM]	FC	EC_50_ [µM]	FC
WT	0.38		0.12	
E21G (0%)	4.7	12	5.4	45
A32V (<0.2%)	3.9	10	0.9	8
F18L (16%)	3.9	10	ND	
E21G/A32V	4.5	12	2.3	19
E21G/A32V/F18L	4.7	12	ND	

(%) reflects the natural occurrence of the respective single NS2A mutations in orthoflaviviruses NS2A proteins as obtained from the Bacterial and viral bioinformatics Research Center (BV-BRC) database (2024). EC_50_: 50% effective concentration; ND: Not determined; FC: Fold change compared to WT.

Next, given that NS2A^L18^ exists naturally in NS2A of some orthoflaviviruses, the single mutants (NS2A^E21G^ and NS2A^A32V^) and a double mutant virus containing (NS2A^E21G/A32V^) were engineered into a DENV-2/EDEN3295 clinical isolate background. Analogous to the mutations in the subgenomic DENV-2/16681 reporter replicon, the single mutants NS2A^E21G^ or NS2A^A32V^ have impact on the viral RNA synthesis or infectious virus production (Fig 4E), and the double mutant exhibited a ~ 2-log_10_ lower RNA synthesis and a ~ 1.3-log_10_ infectious virus production as compared to WT virus (Fig 4F). Analogous to the mutants of the subgenomic DENV-2/16681 reporter replicon, the DENV-2/EDEN3295 NS2A single and double mutant virus were less susceptible to JNJ-1953 (NS2A^E21G^: 45-fold, NS2A^A32V^: 8-fold and NS2A^E21G/A32V^: 19-fold higher EC_50_) compared to the WT virus (Table 4 and S4 Fig). Collectively, these results clearly indicate that the IVRS-identified NS2A mutations are responsible for conferring JNJ-1953 resistance.

### 2.5. JNJ-1953 affects viral RNA synthesis and packaging

**Fig 5:**
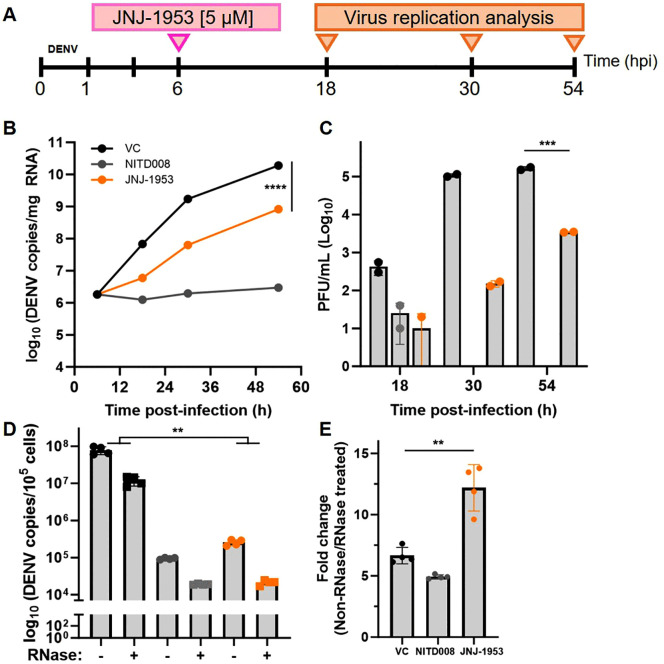
JNJ-1953 reduces viral RNA synthesis and impairs RNA packaging. **A** Schematic. **B,C** Delayed time of drug addition study of JNJ-1953 on DENV-2/EDEN3295 replication. Huh7 cells were infected with DENV-2/EDEN3295 at MOI 1 for 1 h and treated with 5 µM of the respective compounds at 6 h post-infection (hpi). Samples were harvested at the indicated timepoints and subjected to (**B**) intracellular viral RNA quantification by RT-qPCR and (**C**) infectious virus production determined by plaque assay. Data presented in B and C are representative results from two independent experiments with technical readings. The difference in the kinetics of the (B) intracellular viral RNA synthesis and (C) virus titers between virus control (VC) and JNJ-1953 treatment are compared by two-way ANOVA with Geisser-Greenhouse correction. **D,E** Effect of JNJ-1953 on virion assembly. Huh7 cells were infected with DENV-2/Eden3295 at MOI 1 followed by treatment with 5 µM of indicated inhibitors. Cell lysates were harvested 24 hours post infection and subjected to treatment with RNaseA/T1 to remove any unpackaged RNA. (**D**) Intracellular viral RNA quantification of RNaseA/T1 treated and non-treated samples by qRT-PCR. (**E**) Tabulated ratio of the non-RNase treated to RNase treated which provide an indication of the proportion of viral RNA packaged into virions upon treatment with the various inhibitors. Data in D and E are presented as bar graphs showing mean with standard deviation from 2 independent experiments (n = 4). The difference in intracellular viral RNA between DMSO-treated virus control (VC) and JNJ-1953 treatment matched by RNase treatment is compared using two-way ANOVA with Geisser-Greenhouse correction. The difference in the non-RNase to RNase-treated ratio between JNJ-1953 and VC was compared by unpaired Student’s t-test with df = 6. Statistical significances are indicated (** - p < 0.01; *** - p < 0.001; **** - p < 0.0001).

NS2A protein plays a crucial role in various stages of the viral replication cycle, including viral RNA synthesis and virion assembly. To understand the mechanism of action, different approaches were taken. Initially, a delayed time of drug addition study [55] was conducted to assess the effect of JNJ-1953 with respect to intracellular viral RNA synthesis when the cells were treated with 5 µM JNJ-1953 at 6 hours post-infection and monitored for the next 48 hours with sampling at 18, 30 and 54 h post-infection (Fig 5A). In the absence of inhibitor, DENV-2/EDEN3295 infection of Huh7 cells resulted in a rapid increase in intracellular viral RNA until 54 h post-infection as assessed by qRT-PCR. JNJ-1953 reduced viral RNA synthesis by approximately 1–2 log_10_ at different time points (Fig 5B). This reduced intracellular viral RNA led to a corresponding reduction in infectious virus production (Fig 5C). In contrast, treatment with the well-characterized RNA polymerase inhibitor NITD008 showed no net increase in the intracellular RNA overtime that is consistent with no infectious virus production (Fig 5B and 5C). On the other hand, a 1.6- to 2.9-fold (log_10_) at different time points reduction in infectious virus in JNJ-1953 treatment despite the observed increase in net viral RNA synthesis (albeit at a lower level compared to untreated infection control). Next, the effect on viral packaging is evaluated. Hereto, JNJ-1953 treated cells were harvested at 24 h post-infection. The cells were lysed via repeated freeze-thaw cycles followed by treatment with RNaseA/T1 to remove any RNA present within the cell, while shielding those packaged into virions which were then measured using qRT-PCR [56]. Analogous to the delayed time of addition study (Fig 5B), a 2-log_10_ reduction in total intracellular viral RNA is observed compared to DMSO treated cells (Fig 5D), confirming the effect of JNJ-1953 on viral RNA synthesis. Furthermore, the intracellular viral RNA copies before and after RNaseA/T1 treatement were compared and provide an indication of the proportion of packaged viral RNA into virions. Compared to the DMSO and NITD008 controls, the ratio of non-RNase treated to RNase treated cells after JNJ-1953 treatment is significantly higher (*p = 0.0015)*, suggesting an impact of JNJ-1953 on packaging of viral RNA into new virions (Fig 5E). Collectively these results indicate that JNJ-1953 reduces viral RNA synthesis and subsequently impacts the packaging of the viral RNA into newly formed virions consistent with multiple functions of the NS2A protein.

### 2.6. JNJ-1953 interferes with the recruitment of the prM viral structural protein

**Fig 6:**
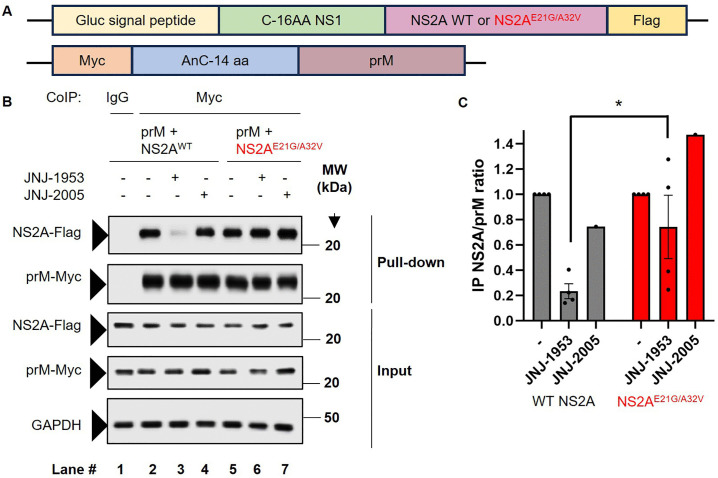
JNJ-1953 destabilizes prM binding to NS2A protein. **A** Schematic showing the construct design of DENV-2 NS2A and prM. The constructs design followed Xie et al. [2] **B** HEK293T cells were transfected with 5 µg each of Myc-prM and Flag-WT or NS2A^E21G/A32V^, followed by treatment with the active JNJ-1953 compound or with its inactive enantiomer, JNJ-2005, at 6 hours post-transfection. Cell lysates were harvested at 44 hours post-transfection and subjected to co-immunoprecipitation using Myc or IgG control antibody. Western blots showing the detection of Flag-NS2A WT and Flag-NS2A^E21G/A32V^ immunoprecipitated with Myc-prM in the absence and presence of JNJ-1953 or JNJ-2005. GAPDH was used as a loading control. **C** Densitometric analysis of the band intensities of WT or NS2A^E21G/A32V^ normalized to prM for the co-immunoprecipitated samples upon treatment with JNJ-1953 or JNJ-2005 compared to untreated. Data is presented as bar graphs showing mean with standard deviation from 4 independent experiments (n = 4) and differences between WT and NS2A^E21G/A32V^ is compared by unpaired Student’s t-test with df = 6 (* - p < 0.05)*.*

Given that the NS2A protein (bound to vRNA) is also reported to interact with viral prM and E proteins to orchestrate viral assembly [2], that the role of JNJ-1953 in affecting the interaction between the NS2A protein and prM was examined. To test this hypothesis, a c-myc prM plasmid was co-transfected with either a Flag WT NS2A, a single mutant NS2A^E21G^ or a double mutant NS2A^E21G/A32V^ plasmid (Figs 6A and S5) into HEK293T cells for co-immunoprecipitation (CoIP) following treatment with 10 µM JNJ-1953 at 6 h post-transfection. CoIP showed that prM specifically pulled-down NS2A protein (Fig 6B; Lane 2), consistent with a previously reported finding [2]. A reduction in the amount of WT NS2A protein pulled down by prM was observed when JNJ-1953 was added during transfection (Fig 6B; Lane 3) and shown by densitometric analysis (Fig 6C), indicating that JNJ-1953 interferes with the interaction between prM and NS2A. Intriguingly, pull down of NS2A^E21G/A32V^ or NS2A^E21G^ by prM was less affected, albeit not significantly, upon JNJ-1953 treatment than that of WT NS2A (NS2A^E21G/A32V^: Fig 6B, lane 6 versus lane 3; Figs 6C, NS2A^E21G^: S5B, Lane 5 vs lane 3; S5C), suggesting that the interaction of the mutated NS2A protein (NS2A^E21G/A32V^ and NS2A^E21G^) with prM is less sensitive to JNJ-1953 (Fig 4B). These experimental results corroborate with the finding that JNJ-1953 is less potent against the NS2A^E21G^ single or NS2A^E21G/A32V^ double mutant viruses (Table 4). We further confirmed the finding by performing a CoIP experiment of prM and WT NS2A interaction by comparing the effect of the active JNJ-1953 with one of its inactive enantiomers, JNJ-2005 (EC_50_ = 5.0 µM; CC_50_ = 8.6 µM). As shown in Fig 6B lane 4, the inactive enantiomer does not affect the interaction of prM with WT NS2A unlike JNJ-1953 (Fig 6C), confirming the specific inhibitory activity of JNJ-1953 on prM-NS2A interaction. It is thus conceivable that in addition to reduced viral synthesis, JNJ-1953 operates via additional, complementary mechanisms, such as impacting viral RNA packaging and affecting the interaction between the proteins prM and NS2A to affect viral assembly, which when combined, provide superior viral inhibition. Such profile would qualify JNJ-1953 as a multimodal inhibitor.

### 2.7. Characterization of drug-like properties of JNJ-1953

JNJ-1953, JNJ-4840 and JNJ-3644 were profiled in several first-line absorption, distribution, metabolism, and excretion (ADME)-Tox assays (Table 3). The compounds are very lipophilic as shown by the high cLogP and chromatographic hydrophobicity index (CHI) log D pH2.6 values. This high lipophilicity seems to be important as NS2A is a highly hydrophobic transmembrane protein located in the ER. JNJ-1953 had good metabolic stability in human liver microsomes (<7.7 µL/min/mg) and moderate stability in mouse liver microsomes (30 µL/min/mg) which was superior compared to the metabolic stability in human and mouse liver microsomes for JNJ-3644 and JNJ-4840 (Table 3). JNJ-1953 showed pH-dependent solubility, with good solubility at pH 2 and lower solubility at physiological pH 7.4 (62 µg/mL). Furthermore, JNJ-1953 showed moderate cytotoxicity in HepG2 cells, which is of comparable magnitude as the cytotoxicity observed in the other tested cell lines (Vero, Huh7) but is accompanied with reasonable selectivity (SI > 4). Further evaluation is needed to assess the effect of JNJ-1953 on drug-induced liver injury or metabolic disruption or if this effect is due to general toxicity of the compound at high concentrations. JNJ-1953 also displayed potential for mitotoxicity based on a cell-based Glu/Gal assay (Glu/Gal ratio = 3.4). Follow up testing using a Seahorse assay is needed to confirm or de-risk the potential of mitochondrial toxicity and evaluation of an on- or off-target effect of JNJ-1953 on the mitochondrial function in nonclinical species is needed. The PK properties of JNJ-1953 following a single intravenous dose of 1 mg/kg (20% HP-β-CD in water) or a single oral administration at 5 mg/kg (PEG400 solution or 20% HP-β-CD in water) were characterized in AG129 mice (Table 5). The compound has low plasma clearance (6.95 mL/min/kg) and a moderate volume of distribution (3.11 L/kg) resulting in a terminal phase elimination half-life T_1/2_ of 7.71 h. Plasma protein binding in mice was high (>99%). The oral bioavailability was moderate (F = 30–72%). In conclusion, although the current ADME-Tox properties are suboptimal, the series provided a new tool compound against a novel target, the NS2A protein.

**Table 5:** *In vivo* pharmacokinetic properties of oral administration of JNJ-1953.

Dose	Vehicle	C_max_ (ng/mL)	AUC_last_ (ng.h/mL)	T_1/2_ (h)	F (%)
5 mg/kg	20% HP-β-CD in water	503	3,739	0.83	30.9
5 mg/kg	PEG400 solution	983	8,661	3.53	71.6

HP-β-CD = 2-hyroxypropyl—β-cyclodextrin; PEG400 = polyethylene glycol 400.

## 3. Discussion

Orthoflaviviruses have a large socio-economic burden of disease. Despite considerable efforts in the past two decades, no clinically approved antivirals are available for orthoflavivirus treatment or prophylaxis, and currently, patients’ options for treatment are limited to measures that solely alleviate symptoms. Therefore, the search for new antivirals targeting endemic, emerging and potential future endemic orthoflaviviruses remains a high priority. Here, we present a novel series of anti-orthoflavivirus small molecule inhibitors targeting NS2A protein, with a novel mechanism of action. Resistance selection and reverse genetics studies pinpointed NS2A protein, a nonenzymatic integral membrane protein [42], with three main functions reported (i) NS2A protein antagonizes the host immune response [30,31,33,36], (ii) NS2A protein functions in viral RNA synthesis within the replication complex [7] and (iii) NS2A protein plays a role in virion assembly [21,29,39], as the molecular target. Attempts to unravel the mechanism of action showed that JNJ-1953 acts as a multimodal inhibitor by affecting the functional roles of NS2A protein in viral RNA synthesis, RNA packaging and virion assembly.

In this paper, a multi-parametric HCI-based HTS approach [43–48,57,58] based on DENV-2 is described to identify novel antiviral candidates active against a wide range of orthoflaviviruses (Fig 1). Compared to single-parametric, cell-based assays which are often used in phenotypic screening campaigns, the multi-parametric readout generated here is used to determine the individual compounds morphological profiles, thereby enabling an early deprioritization of hits with unfavorable mechanism of actions (*e.g.,* targeting the host cell instead of the virus) or undesirable cell phenotypes caused by the compound (*e.g.,* changes in nucleus, cytoplasm, toxicity). This approach maximized at the early stage of screening, the selection of direct-antivirals bocking the virus without any effect on the cell, thereby minimizing the risk of downstream failure, which led to the identification of JNJ-3644 (SIM2NIC: 0.98). This compound was identified with promising antiviral activity against different orthoflaviviruses (Table 1 and Fig 2). Several compounds of the same series as JNJ-3644 were synthesized and JNJ-1953 was selected as lead compound because of its improved features over the other compounds, such as a good solubility at low pH and good metabolic stability in human (<7.7 µL/min/mg) (Table 3 and Fig 2).

An IVRS experiment identified three amino acid substitutions in NS2A – F18L, E21G, A32V – that confer DENV-2 resistance to JNJ-1953 (Fig 4A), suggesting that disruption of NS2A function as the mechanism of action of this compound. NS2A protein has a low sequence identity among all orthoflaviviruses (21–63%), with the highest conserved residues located in the N-terminal half of the protein while functions like viral replication and immune evasion are associated with the full length NS2A protein, the N-terminus of the NS2A protein seems to be critical for viral assembly, particularly the production of infectious particles, through a basic cluster that interacts with other viral components [28,42,59].The three resistance mutations NS2A^F18L^, NS2A^E21G^ and NS2A^A32V^ are situated near or within the conserved N-terminal basic cluster. Residue E21 is highly conserved among the different orthoflaviviruses, except for YFV (V21; Fig 4B). The YFV-17D vaccine strain has known differences in the replication complex compared to WT YFV which may affect antiviral target binding and potency. The NS2A substitutions reported as different between WT YFV (e.g., Asibi) and YFV-17D are not correlating with the resistance mutations observed but locate in the C-terminal domain of NS2A (M118V, T167A, L169F; T172A, S183A) [60]. Hahn *et al*. report that the hydrophobicity profile of the NS2A protein is remarkably conserved and that, provided this profile is maintained, a large number of amino acid substitutions can be tolerated without impairing the function of NS2A protein. No high-resolution structures exist for NS2A protein from any orthoflavivirus, and therefore it remains uncertain whether the YFV-17D NS2A protein within the replication complex differs structurally from YFV NS2A WT. Future testing against YFV WT would be needed in further studies. Based on the DENV-2 IVRS experiment, we postulated that the NS2A^E21^ residue plays a highly important role in the virus replication cycle. Introduction of a glycine on position 21 in DENV-2 (NS2A^E21G^), did not affect the viral RNA synthesis or plaque formation in reverse engineered viruses (Fig 4E and 4F). This was confirmed in ZIKV, where Zhang *et al*. mutated E22 to A, where E22 corresponds to E21 in DENV. The ZIKV E22A mutant replicated similarly as the WT ZIKV, with comparable infectivity and infectious virus production [42]. In contrast, Wu *et al*. concluded that the introduction of alanine substitutions at positions 21–23 in DENV showed a > 1,000-fold reduction in virus yield and an absence of plaque formation [61]. Intriguingly, the combination of the DENV NS2A^E21G^ and NS2A^A32V^ mutations (NS2A^E21G/A32V^) rather than the single mutant viruses (NS2A^E21G^ and NS2A^A32V^) exhibited an attenuated level of viral replication and plaque formation (Fig 4E and 4F), suggesting a synergistic effect of both mutations, underscoring the importance of the N-terminal basic cluster in virus replication. Although resistance against JNJ-1953 is observed in the subgenomic DENV-2/16681 with a single NS2A^F18L^ mutation (Table 4), there was no effect on viral replication (Fig 4C). A leucine is observed in approximately 16% of the orthoflavivirus NS2A proteins and is present in 100% of the DENV-3 serotype strains. Although the resistance mutation was identified during three different IVRS, the fact that the mutation is common in DENV-3 and activity against DENV-3 is observed, the importance of this mutation to acquire resistance will be limited.

NS2A protein is involved in various stages of the viral replication cycle. Here, we showed that by time of drug additions studies that JNJ-1953 is acting on a step after replication is initiated (Fig 3A and 3B). Since the subgenomic DENV-2/16681 replicon construct does not recapitulate the viral entry and virion assembly steps, the observation that the mutants are less susceptible to JNJ-1953 (Table 4) suggests according to the functions ascribed to NS2A protein, a mechanism of action in which viral synthesis is involved. This was confirmed by a 1–2 Log_10_ reduction in intracellular viral RNA. Furthermore, in subsequent experiments, we showed that the addition of JNJ-1953 also impacts viral RNA packaging into newly formed virions (Fig 5E) and diminishes the interaction between NS2A and prM proteins (Fig 6B and 6C), suggesting JNJ-1953 to be a multimodal inhibitor operating via complementary mechanisms. NS2A protein is involved in various processes of the viral replication cycle, with documented roles in viral replication, by interacting with the 3’ untranslated region (3’UTR) of the viral RNA as well as with other viral components involved in virion assembly to coordinate genome encapsulation and assembly [2]. Our data demonstrates that inhibition of NS2A protein by JNJ-1953 affects viral RNA synthesis, the packaging of viral RNA into newly formed virions and the interaction between NS2A and prM proteins. This conclusion is supported by two key observations: (i) JNJ-1953 disrupts the interaction between prM and NS2A protein in a transient transfection system, in the absence of viral infection and other viral proteins, indicating that this effect is not a secondary consequence of impaired RNA synthesis, (ii), JNJ-1953 robustly inhibits viral RNA replication in a replicon system that does not express prM, demonstrating that the compound’s effect on RNA synthesis occurs via a mechanism distinct from its disruption of the prM–NS2A interaction. Nevertheless, given the central role of NS2A protein in viral replication, it remains possible that the observed effects are mediated through direct engagement of NS2A protein by JNJ-1953, that warrants further investigation.

The antiviral orthoflavivirus drug discovery has advanced significantly over the last years. Although there is currently no orthoflavivirus antiviral treatment available, several compounds with different modes of action have been described [62]. The ER supports various steps throughout the whole orthoflaviviral life cycle and provides different opportunities for anti-orthoflaviviral drug development. The transmembrane proteins NS4A and NS4B are considered main drivers in the formation of the replication complex within the ER and were suggested to contribute to membrane rearrangements and stabilize the pore-like opening [63,64]. NS4B has been a well-known target [65], with one molecule, Mosnodenvir, in phase 2 clinical trials targeting Dengue [26]. Mosnodenvir, a highly potent pan-dengue inhibitor blocks the NS3-NS4B interaction within the viral replication complex [66], showed activity *in vivo* in mice and in non-human primates and was found to be safe and well tolerated in phase 1 clinical trials [26]. Drugs targeting NS4A protein are also under evaluation, including compound B and SBI-0090799 which are active *in vitro* against DENV and ZIKV by preventing NS4A protein involvement in replication complex formation [67,68]. To our knowledge, no small molecules have been identified that target NS2A protein, thus highlighting the potential of this series of molecules targeting a novel mechanism of action. In addition, broad-spectrum activity is a desirable feature to prepare for the next orthoflavivirus epidemic, which could emerge from as-yet-unknown or neglected viruses.

In conclusion, our study has successfully identified a series of small molecule inhibitors demonstrating chemical- and virus-dependent activity across orthoflaviviruses. The small molecule inhibitor targets NS2A protein and operates via complementary multimodal mechanisms of reducing viral RNA synthesis, impairing viral RNA packaging and influencing the critical interaction between the NS2A and prM proteins. This finding positions NS2A protein as a promising novel target for future drug development efforts. However, to translate these discoveries into effective therapeutics will require additional studies, notably to provide direct biochemical or biophysical evidence of compound–NS2A protein binding, which is currently lacking. Furthermore, deeper insight into the mechanism of action and the optimization of inhibitor potency, as well as improving ADME-Tox properties will be pivotal for advancing these compounds into clinical development. By laying this groundwork, we open the door to innovative therapeutic strategies that could significantly improve outcomes for patients affected by orthoflavivirus infections, ultimately contributing to global health efforts in combating these pervasive viral threats.

## 4. Methods

### Cells and growth conditions

Adenocarcinoma human alveolar basal epithelial cells (A549; CCL-185, American Type Culture Collection (ATCC)) were cultured in RPMI-1640 (Gibco, Invitrogen Corp.) supplemented with 10% (V/V) fetal bovine serum (FBS, Biowest), 2 mM Ala-Glutamine (Sigma), 25 mM 4-(2-hydroxyethyl)-1-piperazineethanesulfonic acid (Hepes, Sigma) and 0.02 mg/mL gentamicin (Gibco). African green monkey kidney cells (Vero; CL 84113001, European Collection of Authenticated cell cultures (ECACC) and VeroE6; CRL-158, American Type Culture Collection), were cultured in Eagle’s minimal essential medium (MEM; Gibco) supplemented with 10% (V/V) FBS, 2 mM Ala-Glutamine (Sigma) and 0.02 mg/mL gentamicin (Gibco). Vero NS4B-NS5-Tat_LTR-eGFP/hRLuc stable cells, referred to as Vero-GFP, contain a stable expressed NS4B-NS5-Tat and an LTR-eGFP/hRLuc gene. Cells are maintained as described for Vero cells, supplemented additionally with 500 µg/mL geneticin (Gibco) and 200 µg/mL hygromycin (ant-hg-1, InvivoGen). In antiviral assays, the 10% (V/V) FBS is replaced by 2% (V/V) FBS and no hygromycin or geneticin is added. Huh7 hepatoma-derived cells (Sigma) were maintained in Dulbecco’s modified Eagle’s medium (DMEM, Gibco), supplemented with 10% FBS, 2 mM Ala-glutamine, 1 mM sodium pyruvate (Gibco) and 0.02 mg/mL gentamicin. In the antiviral assay, DMEM medium is used supplemented with 10% (V/V) FBS. BHK-21 cells (baby hamster kidney fibroblast cells, ATCC) were cultured in RPMI-1640 medium (Gibco) supplemented with 10% (V/V) FBS and 1% penicillin-streptomycin (P/S). HEK293T (human embryonic kidney, ATCC) cells were maintained in DMEM medium (Gibco) supplemented with 10% (V/V) FBS, 1% P/S and 4.5 g/L glucose. Human monocytic leukemia THP-1 dendritic cell-specific intracellular adhesion molecule-3-grabbing non-integrin (DC-SIGN) cells (TIB-202n, ATCC) were propagated in RPMI-1640 supplemented with 10% (V/V) FBS, 2 mM Ala-Glutamine (Sigma), 25 mM Hepes and 0.02 mg/mL gentamicin. All above-described cells were cultured at 37°C with 5% CO_2_ in a humified incubator. C6/36, an *Aedes albopictus* cell line (ATCC), was maintained in RPMI-1640 medium with 10% (V/V) FBS, 25 mM Hepes and 1% P/S at 28°C in the absence of CO_2_. All cell lines were regularly tested for mycoplasma contamination.

### Virus

The following orthoflavivirus strains and constructs were used in this study: DENV-1/TC974 #666 (National Collection of Pathogenic Viruses (NCPV) 0411281v; GenBank accession: AF180817), DENV-2/16681 (GenBank accession: NC_00174; licensed from Dr. R. Bartenschlager [69]), DENV-2/EDEN3295 (GenBank accession: EU081177 [70]), DENV-2/38865Y10 (obtained through a material transfer agreement with Environmental Health Institute, Singapore), DENV-3/H87 (NCPV 9911281v; GenBank accession: M93130), DENV-4/H241 (NCPV 9910102v; GenBank accession: AY947539), WNV/NY99 (UVE/WNV/1999/US/NY 385–99 (001v-EVA140); GenBank accession: AY842931; European Virus Archive(EVAg)), WNV Uganda B956 (UVE/WNV/1940/UG/UG 956 D117 (001v-EVA1461); GenBank accession: M12294; EvaG), ZIKV/MP1751 (NCPV 1308258v; GenBank accession: KY288905), ZIKV/MR766 (GenBank accession: DQ859059; EVAg), YFV/17D-204 Stamaril vaccine, lot H5105 (GenBank accession: MN708488; Sanofi Pasteur). JEV/SA14-14-2 (GenBank accession: AF315119.1) was generated at KU Leuven using synthetic, overlapping DNA fragments, as described previously [71]. DENV-2/16681-eGFP, carrying an enhanced green fluorescent protein (eGFP) at the amino terminus of the capsid protein, was produced by transfection of in vitro-transcribed RNA of plasmid pFK-DV-G2A into Huh7 cells [69]. This plasmid encodes eGFP and the full-length DENV-2/16681. The infectious cDNA clone pFK-DVs served as the parental construct for cloning of the dengue reporter virus construct DENV-G2A. The reporter gene is followed by the 2A peptide of Thosea asigna virus to liberate the eGFP from the DENV polyprotein during/after translation. The DENV subgenomic reporter replicon (sgDVs-RLuc) consists out of a plasmid (denoted pFK, sgDVsR2A) which contains the non-structural genes NS1-NS5 of the DENV-2/16681 strain and the *Renilla luciferase* (*RLuc*) reporter gene. The sgDVs-RLuc was used to perform a transient DENV replicon assay [69].

### Compounds

A compound library at Johnson & Johnson consisting of 197,135 small-molecule antivirals was used for HCI-based screening. Compound JNJ-3644 and compounds of the same chemical class were synthesized in house. Reference compounds such as, Compound 24 [50], Ribavirin [72], NITD008 [53], 2-CMC [73], JNJ-1A [74], and Brequinar [49] were synthesized in-house. All compounds were > 95% pure, which was confirmed using liquid chromatography-mass spectrometry and proton nuclear magnetic resonance.

### High content imaging (HCI)

For the primary HTS, 1,100 A549 cells/well were seeded in barcoded 384-well carrier plates (Cellcarrier-384, PerkinElmer), the cells were left to adhere at 37°C for 24 hours. Prior to infection, DENV-2/16681-eGFP virus was added to pre-spotted compound 384-well proxiplates (PerkinElmer). After 2 hours, the virus (multiplicity of infection (MOI) 0.15) preincubated with compound (25 µM) was transferred to the carrier plates. After 72 hours of incubation, cells were used for HCI assays. First, live staining was performed by incubating the cells with 50 nM MitoTracker Orange (Thermo Fischer Scientific) for 45 minutes at 37°C. Next, cells were fixed with formaldehyde (2% final concentration; Polyscience) at room temperature for 20 minutes and washed. Plates were then subjected to permeabilization (0.1% Triton-X100). For cell demarcation, nuclei were stained by Hoechst (3.5 µg/mL Hoechst 33258, Invitrogen) and entire cells by HCS CellMask Deep Red (1 µg/mL CellMask Deep red, Invitrogen) (S1A Fig). After staining, plates were imaged on the Cell Voyager 7000 (Yokogawa) confocal microscope, followed by data analysis. HCI data was analyzed with Phaedra HCI analysis software [75]. eGFP positive cells were determined based on the fluorescent signal compared with background signal from the non-infected control cells. The percentage of infected cells was calculated by taking the ratio of infected cells to the total number of cells determined by cell segmentation (S1B Fig). To calculate the percentage of inhibition by compound, the median of the non-infected, DMSO-treated condition (non-infected control) was set to 100% while the median of the infected, DMSO-treated condition was set to 0% inhibition. Cell viability is based on cell count. Assay quality was further assessed using the Z prime for the percentage of GFP fluorescent cells (S1A Fig). The assay was validated using reference compounds with established in vitro activity against DENV-2, including compound 24 [50], Ribavirin [72], NITD008 [53], 2-CMC [73], and JNJ-1A [74] (S1 Fig). The concentration range of the hit confirmation in 4 concentrations was a 4-fold dilution starting at 25 µM (25 – 0.39 µM). The concentration range of the hit confiormation in 10 concentrations was a 3-fold dilution starting at 25 µM (25 – 0.0013 µM).

### SIM2NIC analysis

The similarity to non-infected control (SIM2NIC) analysis quantifies the extent to which the morphology of infected, compound-treated cells resembles the morphology of non-infected cells. A high similarity, with a SIM2NIC close to 1, indicates a direct-acting antiviral. First, 600 features capturing intensity, shape and texture properties at the single-cell level were extracted using all 4 fluorescent channels (Hoechst, HCS CellMaskDeep Red, MitoTracker Orange, eGFP reporter virus) and averaged across all cells per well using a custom-written Acapella (PerkinElmer) image analysis script. Then, features were normalized as z-scores relative to the infected controls/plate. Feature selection was done by data-driven minimum redundancy maximum relevance algorithm as described in Cox *et al.* [43] on all tested compounds with ≥ 2 replicates and ≥ 50% virus inhibition (at any concentration) and resulted in 30 reproducible and non-redundant features, which define the “morphological profile” of each treatment (compound at concentration) and control well. Finally, each morphological profile was compared to the median profile of the non-infected control wells on the same plate by Pearson correlation, resulting in SIM2NIC score. To summarize SIM2NIC at the compound level, the maximum SIM2NIC score overall concentrations at which the compound achieved ≥ 50% virus inhibition and retained ≥ 30% cell count (relative to infected control) was computed and plotted. Compounds with SIM2NIC score >0.8 were considered for further screening.

### Antiviral assays

After hit identification, the antiviral activity was determined against DENV-2/16681-eGFP on three different cell types (Vero, Huh7, and THP-1/DC-SIGN). In brief, 2,500 Vero or Huh7 cells or 7,500 THP-1/ DC-SIGN cells were seeded in 384-well black view plates (Corning, Sigma Aldrich) containing 200 nL of compounds in a 9-fold serial dilution. For Vero and Huh7 cells, the seeded plates were first incubated for 24 h at 37°C, before being infected with DENV-2/16681-eGFP at a MOI of 0.5 (Vero) or 5 (Huh7). THP-1/DC-SIGN cells were infected immediately after seeding the cells with DENV-2/16681-eGFP (MOI 0.5). After three days of incubation at 37°C, viral replication was quantified by measuring eGFP fluorescence using the acumen Cellista (TtpLabtech). The 50% effective concentration (EC_50_) was calculated using concentration-dependent inhibition curves. The cytotoxic effect was determined in the same plates after the eGFP-based readout, except for THP1 cells where toxicity is measured in a non-infected plate. Cell viability or cytotoxicity was measured using an ATPlite cell viability luminescence assay (PerkinElmer), and the luminescence signal was detected with the ViewLux imaging system (PerkinElmer). The 50% cytotoxic concentration (CC_50_) was calculated using concentration-dependent inhibition curves. The selective index (SI) was calculated as the ratio of CC_50_/EC_50_.

The antiviral assays initially used to identify broad-spectrum antiviral inhibitors for JEV, WNV and ZIKV were conducted in a similar way. VeroE6 cells were seeded in a 96-well plate at a density of 1 × 10^5^ cells/well. The next day, a 3-fold (for ZIKV) or 5-fold serial dilution of the compounds was added to the plates. Lastly, the virus was added to the plates (JEV, MOI:0.1; WNV, MOI:0.1; ZIKV, MOI:0.2). After 1 week of incubation in a humified incubator at 37°C with 5% CO_2_, virus-induced cytopathic effect (CPE) was determined by means of the MTS readout method (Promega), as described previously [76]. In the JEV antiviral assay, however, virus-induced CPE was determined using ATPlite, according to the manufacturer’s protocol. The protocol of the YFV antiviral assay was essentially the same as for ZIKV with some differences: cells (Huh7) were seeded at a density of 5,500 cells/well and virus-induced CPE was determined (using MTS) on day 4 post-infection.

Subsequently, inhibitors with activity against different orthoflaviviruses were tested in the antiviral assay using Vero-GFP cells (2,500 cells/well) were seeded in 384-well black view plates (Corning) containing 200 nL of compounds in a 9-fold serially dilution and then placed at 37°C for 24 hours. Next, the cells were infected with the different orthoflaviviruses and corresponding amount of virus (DENV-1/TC974 #666, MOI:1; DENV-2/16681, MOI: 0.5; DENV-3/H87, MOI:0.5; DENV-4/H241, MOI:0.5; WNV/Uganda B956, MOI:0.5; ZIKV/MP1751, MOI:0.16; YFV/17D, MOI:0.06). Three or five days in case of YFV post-infection, the eGFP signal was measured using the acumen Cellista (TtpLabtech). After the eGFP-based readout, the cytotoxic effect was determined in the same plates using ATPlite as described above for Vero cells, except for ZIKV for which the cytotoxic effect was measured on a non-infected plate.

For the testing of the clinical isolate (DENV-2/EDEN3295), Huh7 cells were seeded in a 24-well plate at 1 × 10^5^ cells per well. Cells were first infected with DENV-2/EDEN3295 at a MOI of 0.3 for 1 hour. Virus inoculums were then removed and fresh medium containing the compounds at concentrations ranging from 0.0 1 µM to 50 µM were added. Cells were incubated for additional 48 hours at 37 °C and the supernatants were collected. Virus titers in the supernatants were determined by standard plaque assay on BHK-21 cells. Standard plaque assay on BHK-21 was performed as previously described [77]. EC_50_ values were determined using a sigmoidal dose response (variable slope) non-linear regression model in GraphPad Prism software. For antibody-dependent enhanced (ADE) infection, DENV-2/38865Y10 infection at MOI 10 and humanized 4G2 (0.05 μg) were mixed and incubated on ice for 1 hour to allow the formation of immune complexes. THP-1 cells (1 × 10^5^) were infected with the immune complexes for 2.5 hours at 37 °C with shaking. Cells were then washed once with phosphate-buffered saline (PBS) before resuspending in RPMI-1640 medium containing the compound at concentrations ranging from 0.1 nM to 25 µM followed by a further incubation of 48 hours. After 48 hours, supernatants were harvested and subjected to virus titer determination by standard BHK-21 plaque assay.

### Tetravalent duplex real-time quantitative polymerase chain reaction (RT-qPCR)

The tetravalent antiviral RT-qPCR was based on as previously described protocol [78]. In brief, Vero cells (10,000 cells/well) were seeded in 96-well plates containing a serial dilution of the test compound. 24 Hours after seeding, the cells were infected with DENV (DENV-1/TC974 #666, MOI:0.1; DENV-3/H87, MOI:0.025; DENV-4/H241, MOI:0.6) and incubated at 37°C for three days. Intracellular RNA was measured by washing the adherent cells of the plates without supernatant with cold PBS and plates were incubated at -80°C for at least 24 hours. After 24 hours the cells were lysed with Cells-to-CT Bulk Lysis Reagents kit (Thermo Fisher Scientific) and the cell lysates were used to prepare cDNA (using Expand Reverse Transcriptase) of the target sequences, the 3’-untranslated region (3’UTR) of DENV (Forward primer: 5′-GGCCAGGTCATCACCATT-3′, Reverse primer: 5′-GAGACAGCAGGATC TCTGGTC-3′, Probe: FAM-5′-AAGGACTAGAGGTTAGAGGAGACCCCCC-3′-BHQ1), and the cellular housekeeping reference gene β-actin (Forward primer: 5′-GGCCAGGTCATCACCATT-3′, Reverse primer: 5′-ATGTCCACGTCACACTTCATG-3′, Probe: HEX-5’-TTCCGCTGC(ZEN)CCTGA GGCTCTC-3IABkFQ). Subsequently, a duplex RT-qPCR was performed on a Lightcycler480 II instrument (Roche) at the following conditions: 10 minutes at 95°C, followed by 40 cycles of 10 seconds at 95°C, 1 minute at 60°C.

### Time-of-addition assay

The time-of-addition (TOA) assay was performed as shown in the schematics of Fig 3 [79]. Briefly, 1 × 10^5^ Huh7 cells were infected with DENV-2/EDEN3295 at MOI 1 for 1 hour followed by treatment with 5 µM JNJ-1953 at 1, 2, 4, 6, 10 and 19 h post-infection (post-treatment). For pre-treatment, the cells were exposed to 5 µM of the respective compounds for 2 hours prior to infection and subsequently replaced by medium without compound. For co-treatment, the cells were infected with the virus that was mixed with 5 µM of the compounds for 1 hour and subsequently replaced with media. The endpoint assessment is by quantifying the infectious virus production after 24 hours post-infection. Supernatants were harvested at the indicated timepoints and subjected to plaque quantification by plaque assay and extracellular viral RNA quantification by real time RT-PCR.

### DENV-2/16681 in vitro resistance selection

Vero cells were seeded at a density of 1 × 10^5^ cells/well in a 96 well plate. The next day, cells were infected (MOI of 0.1) with DENV-2/16681 and incubated for 96 hours at 37°C in the presence of a 2-fold serial dilution of JNJ-3644 (5-0.039 µM) for 4 days at 37°C. After 4 days, cells were microscopically checked for CPE, and the supernatant from two adjacent wells showing 30–70% CPE was collected and pooled. The collected supernatant was subsequently used to infect freshly seeded cells. The remaining supernatant was stored at - 80°C until further analysis. Virus was passed twice a week. During passage of the virus, the start concentration of the compound gradually increased. This procedure was repeated until the observed EC_50_ value approached the cytostatic concentration of the compound. To check for spontaneous and/or tissue-culture-adapted mutations, part of the wells served as WT virus controls to which no compound was added. WT DENV-2/16681 was passed using Vero cells in a similar way to compound-treated virus. Further analysis includes NGS of the viral RNA isolated from cell culture supernatant (140 µL) using a QIAamp Viral RNA Mini kit (Qiagen) per the manufacturer’s protocol. Viral RNA was amplified into double-stranded DNA using a NuGEN Trio RNA-Sequence kit per manufacturer’s protocol. Full DENV-2/16681 genome was sequenced using NGS technology (Illumina). Sequences were filtered for viral content by aligning the reads to DENV-2/16681 viral genome (GenBank accession: NC_00174). A coverage cut-off value of 100 and a 15% read frequency cut-off were used for the reliable detection of amino acid variants.

### Transient DENV replication assay

Mutant subgenomic DENV reporter replicons (sgDVs-RLuc) each containing a NS2A single, double or triple resistance mutation (Epoch Life Science) were used to determine the compound resistance imposed by each of the mutations. First, each resistance mutation was inserted separately into the sgDVs-RLuc replicon. The plasmid (denoted pFK-sgDVs-R2A) contains the non-structural genes *NS1-NS5* of the DENV-2/16681 strain with cell-adaptive mutations in *NS3* (A546V and H451P), *NS4A* (I116M) and *NS5* (E892K), and the *Renilla luciferase* (RLuc) reporter gene [69]. The plasmids of both wild-type and mutant sgDVs-Rluc were used to produce *in vitro* transcribed (IVT) DENV RNA as described previously and electroporated into Huh7 cells [69]. In brief, plasmid DNA was linearized with XbaI (located at the end of the 3’ untranslated region of the viral genome). *In vitro* transcription was performed with a mMessage mMachine SP6 kit (Ambion) according to the manufacturer’s protocol. The molecular mass and integrity were checked by agarose gel electrophoresis. IVT RNA of both WT and mutant sgDVs-R2A was transiently transfected into Huh7 cells. To this end, 10 µg IVT linear RNA was electroporated into a cell suspension containing 4x10^6^ Huh7 cells (electroporation at 975 µF and 270 V; Gene Pulser II, Bio-Rad). Transfected cells were seeded on plates containing the compound at concentrations ranging from 0.04 µM to 25 µM and incubated at 37°C. At 48 hours after transfection, viral replication was quantified by measuring the RLuc activity using the Renilla-Glo Luciferase assay system (Promega) following the manufacturer’s instructions and detected using the ViewLux..Full-length DENV-2/EDEN3295 cDNA clone (GenBank accession: EU081177) used in this study has been previously described [80]. Site-directed mutagenesis was used to generate the different NS2A mutations (single mutants E21G or A32V and the double mutant E21G A32V) on the full-length DENV-2/EDEN3295 clone. IVT RNA was obtained from the full-length cDNA clone using T7 mMESSAGE mMACHINE kit (Ambion).

IVT RNA of both WT and mutant DENV-2/Eden3295 virus was transiently transfected into C6/36 cells. To this end, 10 µg IVT linear RNA was electroporated into a cell suspension containing 1x10^7^ C6/36 cells/mL (electroporation at 25 µF and 850 V,2 pulses with an interval of 3 s) [80]. Electroporated cells were seeded (3 x 10^5^) into 12-well plates and incubated at 37°C in the presence of 5% CO_2_. Supernatants from the transfected C6/36 cells were collected on day 7 after transfection (P0 virus) and passed once in C6/36 to obtain the P1 virus stock for compound efficacy evaluation assays. The iIVT RNAs of DENV-2 WT or the various NS2A mutants were transfected into BHK-21 cells as previously described [80] to profile its replication kinetics over a course of 3 days. Supernatants were collected for infectious virus quantification by standard plaque assay while the transfected cells were washed once prior to lysing with RLT buffer (Qiagen) for intracellular viral RNA quantification by RT-qPCR.

### RNase treatment of JNJ-1953 treated cells

Huh7 cells were infected with DENV-2/EDEN3295 (MOI 1) for 1 hour and treated with 5 µM. 24 Hours after infection cells were harvested and lysed by performing two freeze-thaw cycles. The lysates were divided into two equal aliquots and to one set of the aliquots, 1 U of RNase A/T1 Cocktail Enzyme Mix (Ambion) was added to degrade any host and unpackaged viral RNA. After incubation for 30 min at 37°C, viral RNA extraction (QIAamp Viral RNA Mini Kit, Qiagen) and qRT-PCR was performed to quantify the amount of packaged viral RNA in the cells [56].

### Construction of DENV-2 prM and NS2A mammalian expression plasmids

The mammalian expression plasmids of DENV-2/EDEN3295 (EU081177) prM and NS2A were designed and constructed as described in [2] (see schematic in Fig 5A). Briefly, the signal peptide from *Gaussia luciferase (Gluc)* together with the last 16-amino acids (aa) of NS1 is fused to the N-terminus of NS2A to ensure correct processing and membrane topology of NS2A. This NS2A construct (WT) is then cloned into a C-terminal Flag-tagged pcDNA3.1 + vector (Genscript) using *Nhe*I and *Xho*I restriction sites. The WT NS2A plasmid was subjected to reverse-engineering of the E21G single (NS2A^E21G^) and E21G/A32V double mutations (NS2A^E21G/A32V^) using Quikchange II XL site-directed mutagenesis kit (Stratagene) according to manufacturer’s instructions. For the prM construct, the anchor C signal peptide (AnC-14aa) is retained at the N-terminus of prM to ensure correct targeting to the ER membrane. This prM construct is then cloned into a N-terminal Myc pcDNA3.1 + vector (Genscript) with *Hind*III and *Xho*I restriction sites.

### Co-immunoprecipitation, SDS-page and western blot

The co-immunoprecipitation was performed as described in [2]. Briefly, single, or various combinations of expression plasmids carrying WT NS2A, NS2A^e21G^ or NS2A^E21G/A32V^ and prM (each at a concentration of 5 µg) were transfected into HEK293T cells (2.5 × 10⁵ cells per 10-cm dish) using the Fugene 6 transfection reagent (Promega). At 6 hours post-transfection, the cells were treated with 10 µM of JNJ-1953 or its inactive enantiomer (JNJ-2005). Following a 44-hour incubation period, the cells were lysed in 0.5 mL immunoprecipitation (IP) buffer (20 mM Tris, pH 7.5, 100 mM NaCl, 0.5% DDM, and EDTA-free protease inhibitor cocktail [Roche]) with rotation at 4°C for one hour. The lysates were then clarified by centrifugation at 21,000 × g and 4°C for 30 minutes. The supernatants (200 µL) were combined with IP buffer (20 mM Tris [pH 7.5], 0.5% DDM), and NaCl was added to achieve a final concentration of 400 mM. 2 µg of rabbit anti-c-myc antibody (Sigma) were then added to the mixture, followed by agitation overnight at 4°C (end-to-end shaker) to form immune complexes. The immune complexes were captured by the addition of 30 μL of Pierce Protein A/G Plus Agarose (Thermo Scientific), and the mixtures were tumbled further for 1–2 hours. Thereafter, the beads-bound immune complexes were collected by centrifuging at 900 × g at 4°C for 3 min and washed five times with PBS containing 0.1% Tween 20 (PBS-T). The beads-bound immune complexes were eluted by boiling in 5 × sodium dodecyl sulfate (SDS) (Bio Basic Asia Pacific) sample buffer supplemented with 50 mM dithiothreitol (DTT) at 100°C for 10 min. The tubes were briefly vortexed and then subjected to centrifugation at 10,000 × g for one minute. A total of 40 μL of the sample was loaded onto a 4 – 20% SDS-PAGE gel (Bio-Rad, Cat # 4561094), including a precision Plus protein standard dual color (Bio-Rad, Cat # 1610374). Subsequently, the proteins were resolved and transferred onto a nitrocellulose membrane (Bio-Rad) using the Bio-Rad Blotting System. To prevent the light chain (size 26 kDa) from obscuring the prM or NS2A protein bands (size approx. 24 kDa), the protein blot was stained with Ponceau as previously described [81] and trimmed precisely at 25 kDa. Subsequently, the blot was incubated for 1 h in a blocking buffer containing 5% skim milk in PBS-T. The blot was washed twice with PBS-T and incubated with a primary antibody, either rabbit anti-Myc (Sigma) or mouse anti-Flag (Sigma), and an anti-GAPDH (Thermo Fisher) or anti-tubulin (Sigma), for approximately 16 hours at 4°C while shaking. Following three washes with PBS-T buffer, the protein blot was incubated with horseradish peroxidase (HRP)-conjugated rabbit or mouse antibody for one hour at room temperature on a shaker. To demonstrate equal expression and loading, the protein blot with inputs (10%) was probed with an anti-Myc, anti-Flag, or anti-GAPDH or anti-tubulin antibody. Subsequently, the blots were subjected to three comprehensive washes with PBS-T buffer. Thereafter, the ECL substrates (Advansta Inc.) were applied in accordance with the instructions provided. Subsequently, the chemiluminescence signals were detected using the ChemiDoc system (Bio-Rad).

## Supporting information

S1 Text Supplementary material and methods. (DOCX)

S1 Fig High-content imaging DENV-2/16681-GFP antiviral assay in A549 cells. **A** Representative image of DENV-2/16681-GFP infected A549 cells. 10 x magnification images from CV7000 Yokagawa showing eGFP signal coming from the DENV-2/16681-GFP infection (eGFP, green), Hoechst staining (nuclei, blue), MitoTracker orange staining (mitochondria, Orange) and Cell-Mask Deep red staining (cytoplasm and nucleus, Red). Scale bar: 50 µM. **B** Reproducibility and robustness of the assay. Percentage of infection (top), cell count (middle) and Z-prime (bottom) for each High throughput screening (HTS) run. The complete primary screen was divided into 6 different runs. Z’ factors (>0.7 for most plates) were consistent across the different runs. **C** pIC_50_ values (negative log of the 50% inhibitory concentration) for 5 standard used reference compounds (Ribavirin, 2’CMC, JNJ-1A, NITD008 and Compound 24). The pIC50 value for each reference compound is determined each HTS run. The potency of the reference compounds across the different assay runs was reproducible, demonstrating assay robustness.
(DOCX)

S2 Fig Antiviral activity of JNJ-3644 series in different assays against different orthoflaviviruses. **A** Antiviral activity of JNJ-3644 and the seven other isomers against DENV-2/16681 in Vero cells based on GFP expression. **B** Tetravalent dengue antiviral assay (DENV-1, DENV-2, DENV-3 and DENV-4) of JNJ-3644 in Vero cells using RT-qPCR as readout. **C** Antiviral activity of JNJ-3644 (green), JNJ-4840 (purple) and JNJ-1953 (orange) in Vero-GFP cells against different orthoflaviviruses (DENV-1, DENV-2, DENV-3, DENV-4, WNV, YFV and ZIKV). **D** Antiviral activity of JNJ-1953 against ZIKV H/PF/2013 in Huh7 cells based on plaque forming units per mL.
(DOCX)

S3 Fig Replication properties of resistant subgenomic replicons. **A** Schematic representation of the subgenomic DENV-2/16681 reporter replicon sgDVs-R2A [69]. **B** Effect of resistance mutations in NS2A on replication fitness. Resistance mutations identified in Fig 4A were introduced into sgDVs-R2A. Huh7 cells were transfected with 10 µg in vitro transcribed RNA of Wild-type (WT) or mutant sgDVs-R2A and lysed at 48 h post-transfection. Renilla luciferase activity was measured as marker of replication. Relative light units (RLU). Plotted are the mean ± SD from two independent experiments (in duplo), each carried out with independent RNA preparations.
(DOCX)

S4 Fig Plaque reduction of the different DENV-2/Eden3295 mutant viruses (E21G, A32V and E21G/A32V). Viral inhibition of the different mutant DENV-2/Eden3295 strains at two concentrations (0.2 and 2 µM) of JNJ-1953. At the highest concentration, JNJ-1953 treatment induces ~40–45% virus reduction for the NS2A mutant viruses, while >99% reduction is observed for the WT DENV-2/EDEN3295 virus strain.
(DOCX)

S5 Fig JNJ-1953 destabilizes prM binding to NS2A. **A** Schematic showing the construct design of DENV-2 NS2A and prM. The constructs design followed Xie *et al.* [2] **B** HEK293T cells were transfected with 5 µg each of Myc-prM and Flag-WT, NS2A^E21G^ or NS2A^E21G/A32V^, followed by treatment with the JNJ-1953, at 6 hours post-transfection. Cell lysates were harvested at 44 hours post-transfection and subjected to co-immunoprecipitation using Myc or IgG control antibody. Western blots showing the detection of Flag-NS2A WT, FLAG-NS2A^E21G^ and Flag-NS2A^E21G/A32V^ immunoprecipitated with Myc-prM in the absence and presence of JNJ-1953. Tubulin was used as a loading control. **C** Densitometric analysis of the band intensities of WT, NS2A^E21G^ or NS2A^E21G/A32V^ normalized to prM for the co-immunoprecipitated samples upon treatment with JNJ-1953 compared to untreated. Data are presented as bar graphs showing mean with standard deviation from 3 independent experiments.
(DOCX)

S1 Table Activity of JNJ-3644 different enantiomers against DENV-2/16681 in Vero cells. (DOCX)

S2 Table Antiviral activity of JNJ-3644 against other RNA and DNA viruses. (DOCX)

S3 Table Percentage of allele frequency of the mutations developed in the DENV-2/16681 strain at passage 13 following an in vitro resistance experiment using JNJ-3644. (DOCX)

S4 Table Percentage of allele frequency of the mutations developed in the DENV-2/16681 strain at passage 18 following an in vitro resistance experiment using JNJ-1953. (DOCX)

S5 Table Percentage of allele frequency of the mutations developed in the DENV-2/16681 strain at passage 18 and passage 28 following an in vitro resistance experiment using JNJ-1953. (DOCX)

S6 Table Natural occurrence of the NS2A mutation residues in clinical isolates. (DOCX)

S7 Table Antiviral activity of JNJ-3644 and JNJ-4840 against WT and NS2A mutant DENV-2 subgenomic constructs. (DOCX)
