# Supplementary material for: An orthoflavivirus inhibitor targeting multifunctional NS2A protein, a previously unidentified target
Source: PLoS Pathog. 2026 May 5;22(5):e1014190. doi: 10.1371/journal.ppat.1014190 (PMC13166939; doi:10.1371/journal.ppat.1014190)
Supplement: S1 Text — (DOCX) [file ppat.1014190.s001.docx]

Material and Methods

**Additional cell lines and viruses – antiviral assays**

The HeLa cell line (Cervical epithelial, human, CCL-2) was obtained from American Type Culture Collection (ATCC; Manassas, VA, USA) and was cultured for infection experiments in Roswell Park Memorial Institute Medium (RPMI)-1640 medium supplemented with 10% heat-inactivated Fetal bovine serum (FBS), 25 mM Hepes, 100 mM L-glutamine and 0.02 mg/mL gentamycin. In the medium for the antiviral assay, the 10% heat-inactivated FBS is replaced by 2% heat-inactivated FBS. HepG2.117 cells were kindly provided by Prof. M. Nassal, University Hospital Freiburg, Freiburg, Germany (1). HepG2.117 cells were cultured in [Dulbecco's Modified Eagle Medium](https://www.google.com/search?sca_esv=a1588e28351376e1&rlz=1C1GCEA_en__1161__1161&sxsrf=AE3TifMFXb1SRuOohIRjJUdpd2DiQsNNoA%3A1757500810422&q=Dulbecco%27s+Modified+Eagle+Medium&sa=X&ved=2ahUKEwiGtP3bgM6PAxXr9wIHHeVrKf4QxccNegQIIhAB&mstk=AUtExfC3u7UMIE0B5O2LR4MOYBeDvP-kdef60oSaiEqwiN2cNrY4Yyy7XM1mS5svgJN477hY43vkxwZzlg-jkJiXsKaGR1omjmwNyPujXt9XGAVz25OYAwgAZtFix-t4aoPWErr_tODeNHldRE6eJPUPSbNRivvpYJL71v7X7-oCPfC67KThLPWt1kEcsz3-WaPy7ixvZ7VLU4BGkjS6cHttIfnbwnoYz1N-0Hse7LI9Qxp4Jmtu6wcoujG8lfjBU4L9ISf6kIBx-W_HijHa52JFUqkX&csui=3) (DMEM) supplemented with 10% FBS, 2 mM L-glutamine, 0.02 mg/mL gentamicin, 0.080 mg/mL hygromycin (Roche), 0.500 mg/mL geneticin (G418; Gibco), and 100 ng/mL doxycycline (Sigma). HepG2.117 is an inducible hepatitis B virus (HBV)-replication cell line (1). It was established by introduction of a tetracycline (Tet)-responsive element (TRE)-controlled HBV genome (Genotype D, Subtype ayw) expression vector (pTRE-HBVT) into a HepG2 cell line that stably expresses a Tet responsive trans-activator (tTA). HBV pregenomic RNA is transcribed under the control of the TRE-controlled minimal cytomegalovirus (CMV) promotor upon doxycycline removal from the culture medium, leading to capsid assembly and DNA synthesis. Huh7-Luc cells (Huh7 human hepatoma cells that are stably transfected with a selectable self-replicating subgenomic hepatitis C virus [HCV] Genotype 1b [Clone ET] RNA sequence harboring a luciferase [Luc] reporter gene) and Huh-7-CMV-Luc (Huh7 cells containing a CMV major immediate early promoter – Luc construct) were obtained from Heidelberg University Hospital (Heidelberg, Germany) (2). Both cell lines were cultured in DMEM supplemented with 10% heat-inactivated FBS, 0.02 mg/mL gentamycin, 2 mM L-glutamine, and 0.25 mg/mL G418. The same medium was used in the antiviral assay. LLC-MK2 (Monkey kidney cells, CCL7) obtained from ATCC, were grown in DMEM supplemented with 10% heat-inactivated FBS, 2 mL L-glutamine, 1 mM nonessential amino acids, penicillin (100 IU/mL), streptomycin (0.100 mg/mL), and 1 mM sodium pyruvate. All cells were incubated at 37°C in the presence of 5% CO_2_. Recombinant human rgRSV224[63](https://www.nature.com/articles/s41467-017-00170-x#ref-CR63) was licensed from the National Institutes of Health (Bethesda, MD, USA) and propagated in HeLa cells (3). Chikungunya virus (CHIKV)/S27 was obtained through the European Collection of Authenticated Cell Cultures (ECACC; Cat. No: 0006254v). Recombinant hMPV A2 -GFP (CAN97-83) strain (M121-; ViraTree; USA), harboring an eGFP reporter in their genome was licensed from the National Institutes of Infectious Diseases (Tokyo, Japan) (4). INF A/Taiwan/1/1986 (H1N1, TW) or INF B/Singapore/222/1979 (Yamagata lineage) are available in-house. Zika Virus (ZIKV) H/PF/2013 (GenBank accession: KJ776791; Evag).

**Antiviral assays**

The antiviral activity was determined against other DNA and RNA viruses. The respiratory syncytial virus (RSV) antiviral assay was described in Roymans et al. (5). In brief, HeLa cells (3,000 cells/well) are infected with RSV-eGFP at multiplicity of infection (MOI 1) and incubated at 37°C for 3 days in 384-well black microtiter plates with a serial dilution of the compound. The produced eGFP fluorescence is measured using the Envision (PerkinElmer). The level of eGFP expression in infected cells correlates with the level of viral replication. In parallel, possible effects on the metabolic state of the cells (e.g. cytocidal or cytostatic) are determined using an ATP-based bioluminescent readout. The antiviral activity against CHIKV/S27 (Togaviridae) was measured with a cytopathic effect (CPE) inhibition assay using ATPLite (PerkinElmer). This assay readout is based on the bioluminescent measurement of adenosine triphosphate (ATP) in metabolically active cells. Briefly, Huh7 cells (8,000 cells/well, CHIKV) were seeded in 384-well blackview plates (Costar) containing serially diluted test compound in cell culture medium (DMEM supplemented with 2% FBS) and were infected with CHIKV/S27 at a MOI of 0.25. Plates were incubated at 37°C and 5% CO_2_ for 2 days until the viral CPE in the virus control wells reached ~100%/. Then, ATPLite was added to all wells to assess the viability of the cells and thus the preventive effect of the antiviral test compound on CPE. Luminescence was measured using ViewLux (PerkinElmer). In parallel, cytotoxicity was assessed in non-infected Huh7 cells using the same ATP-based bioluminescent readout. The antiviral activity against Influenza (INF) A and INF B (Orthomyxoviridae) was determined using a neuraminidase activity. A549 cells (6,000 cells/well) were infected with the INF A/Taiwan/1/1986 (H1N1, TW) or INF B/Singapore/222/1979 (Yamagata lineage) at an MOI 0.05 in 384 well plates. Plates were incubated at 37°C and viral infection is measured after 2 days. The neuramidase activity is determined on the cells using 2’-(4-Methylumbelliferyl)-a-D-N-acetylneuraminic acid at 25 µM as a fluorescent substrate. Viral neuramidase converts the substrate into a fluorometric product which is directly proportional to the neuraminidase activity of the viral sample. The readout is done using a fluorescence microplate reader (Fluoroskan, Thermo Scientific). In parallel, the toxicity of the compounds is tested in A549 cells and is measured using the same ATP-based bioluminescent readout as for CHIKV. 20,000 HepG2.117 cells per well were plated into 96-well plates 1 day before compound addition. During the antiviral testing, cells were cultured in DMEM medium with 1% nonessential amino acid solution and 1% L-glutamine in the absence of doxycycline and presence of 2% FBS. At the end of the drug treatment, the supernatant was removed, and intracellular total DNA was extracted. Briefly, 100 µL of a 0.33% NP-40 solution was added per well, the plate was incubated at 4°C for 5 minutes and spun at 1,500 revolutions per minute (rpm) for 5 minutes to remove cell debris. Of the lysate, 35 µL was added to 65 µL QuickExtract DNA Extraction solution 1.0 (Epicentre) in a 96-well PCR plate and the plate was incubated for 6 minutes at 65°C and 2 minutes at 98°C in a PCR machine. Of the extracted total DNA, 10 µL was used for the quantification of HBV DNA in a RT-qPCR assay. The Δ cycle threshold (Ct) method was used to calculate 50% effective concentration (EC_50_) values. Toxicity was evaluated on HepG2 117 cells (20,000 cells/well) seeded in 96-well culture plates (Nunc) in RPMI-1640 medium with 2% L-glutamine in the presence of 2% FBS containing serially diluted test compound in cell culture medium and were incubated for 4 days. Cytotoxicity is measured using the ATP-based bioluminescent readout. The antiviral activity against HCV (Flaviviridae) was tested in an HCV replicon-containing cell culture system. In brief, Huh7-Luc replicon-containing cells were seeded in 384-well plates (2,500 cells/well) and incubated for 3 days with a concentration range of serially diluted compound in cell culture medium without G418. HCV replicon RNA replication was determined by means of measuring the firefly luciferase reporter gene expression using the SteadyLite Plus assay kit (PerkinElmer) and luminescent measurement using a ViewLux reader (PerkinElmer). A toxicity assay was performed using the Huh7-CMV-Luc cells (containing an hCMV-MIEP-Luc construct). Cells were seeded in 384-well culture plates (2,500 cells/well) in cell culture medium without G418 and incubated for 3 days at 37°C in the presence or absence of the compound. Luciferase activity was quantified using the SteadyLite^+^ assay kit and luminescent measurement using a ViewLux reader. LLC-MK2 cells (6,000 cells/well) are infected with hMPV A2 -GFP (CAN97-83) at MOI 0.1 in Opti-MEM reduced serum medium without FBS, containing 0.5 µg/mL trypsin and incubated at 37°C with compound for 3 days. The produced eGFP fluorescence is measured using the Acumen. The level of eGFP expression in infected cells correlates with the level of viral replication. In parallel, possible effects on the metabolic state of the cells (e.g. cytocidal or cytostatic) are determined using an ATP-based bioluminescent readout. The antiviral activity against ZIKV was determined in a Huh7 infection model. Briefly, 1 × 10^5^ Huh7 cells were infected with ZIKV H/PF/2013 (GenBank accession: KJ776791) at MOI 0.5. The infected cells were subsequently treated with the indicated concentrations of JNJ-1953 for 24 hours. Supernatants were harvested at 24 hours post-infection and subjected to standard baby hamster kidney (BHK)-21 plaque assay to assess the viral plaque forming units (PFU) at various treatment concentrations. The efficacy of JNJ-1953 was determined by sigmoidal dose response (variable slope) non-linear regression in GraphPad Prism software.

**BHK-21 plaque assay**

A monolayer of BHK cells were cultured to approximately 80% confluency in 24-well plates (NUNC). Serial dilutions (10^−1^ to 10^−8^) of the virus stock in RPMI 1640 (200 μL of each dilution) were used to infect BHK-21 monolayers. Following incubation at 37°C and 5% C0_2_ for 1h with gentle rocking at 15 min intervals, the medium was decanted and 0.5 ml of 1% (w/V) carboxymethyl cellulose in RPMI supplemented with 2% FBS was added to each well. After 5 days incubation, the cells were fixed with 4% paraformaldehyde and stained with 1% crystal violet dissolved in phosphate buffered saline (PBS). After thorough rinsing with water, the plates were dried and the plaques were scored visually.

1. Sun D, Nassal M. Stable HepG2- and Huh7-based human hepatoma cell lines for efficient regulated expression of infectious hepatitis B virus. J Hepatol. 2006;45(5):636-45.

2. Lohmann V, Korner F, Koch J, Herian U, Theilmann L, Bartenschlager R. Replication of subgenomic hepatitis C virus RNAs in a hepatoma cell line. Science. 1999;285(5424):110-3.

3. Hallak LK, Spillmann D, Collins PL, Peeples ME. Glycosaminoglycan sulfation requirements for respiratory syncytial virus infection. J Virol. 2000;74(22):10508-13.

4. Shirogane Y, Takeda M, Iwasaki M, Ishiguro N, Takeuchi H, Nakatsu Y, et al. Efficient multiplication of human metapneumovirus in Vero cells expressing the transmembrane serine protease TMPRSS2. J Virol. 2008;82(17):8942-6.

5. Roymans D, Alnajjar SS, Battles MB, Sitthicharoenchai P, Furmanova-Hollenstein P, Rigaux P, et al. Therapeutic efficacy of a respiratory syncytial virus fusion inhibitor. Nat Commun. 2017;8(1):167.
