# Supplementary material for: An orthoflavivirus inhibitor targeting multifunctional NS2A protein, a previously unidentified target
Source: PLoS Pathog. 2026 May 5;22(5):e1014190. doi: 10.1371/journal.ppat.1014190 (PMC13166939; doi:10.1371/journal.ppat.1014190)
Supplement: S1 Fig — A Representative image of DENV-2/16681-GFP infected A549 cells. 10 x magnification images from CV7000 Yokagawa showing eGFP signal coming from the DENV-2/16681-GFP infection (eGFP, green), Hoechst staining (nuclei, blue), MitoTracker orange staining (mitochondria, Orange) and Cell-Mask Deep red staining (cytoplasm and nucleus, Red). Scale bar: 50 µM. B Reproducibility and robustness of the assay. Percentage of infection (top), cell count (middle) and Z-prime (bottom) for each High throughput screening (HTS) run. The complete primary screen was divided into 6 different runs. Z’ factors (>0.7 for most plates) were consistent across the different runs. C pIC50 values (negative log of the 50% inhibitory concentration) for 5 standard used reference compounds (Ribavirin, 2’CMC, JNJ-1A, NITD008 and Compound 24). The pIC50 value for each reference compound is determined each HTS run. The potency of the reference compounds across the different assay runs was reproducible, demonstrating assay robustness. (DOCX) [file ppat.1014190.s002.docx]

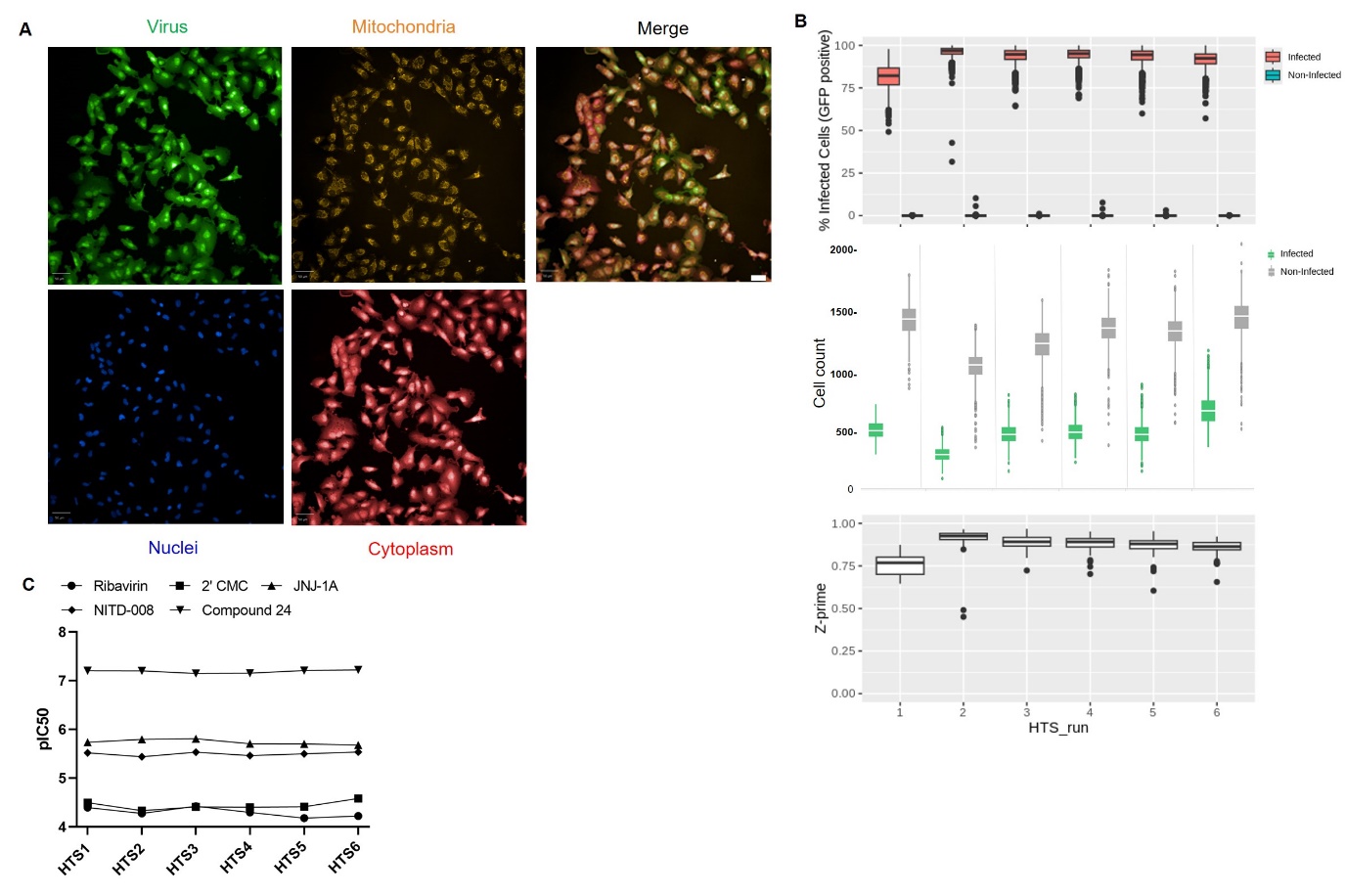


**S1 Fig.: High-content imaging DENV-2/16681-GFP antiviral assay in A549 cells. A** Representative image of DENV-2/16681-GFP infected A549 cells. 10 x magnification images from CV7000 Yokagawa showing eGFP signal coming from the DENV-2/16681-GFP infection (eGFP, green), Hoechst staining (nuclei, blue), MitoTracker orange staining (mitochondria, Orange) and Cell-Mask Deep red staining (cytoplasm and nucleus, Red). Scale bar: 50 µM. **B** Reproducibility and robustness of the assay. Percentage of infection (top), cell count (middle) and Z-prime (bottom) for each High throughput screening (HTS) run. The complete primary screen was divided into 6 different runs. Z’ factors (>0.7 for most plates) were consistent across the different runs. **C** pIC_50_ values (negative log of the 50% inhibitory concentration) for 5 standard used reference compounds (Ribavirin, 2’CMC, JNJ-1A, NITD-008 and Compound 24). The pIC_50_ value for each reference compound is determined each HTS run. The potency of the reference compounds across the different assay runs was reproducible, demonstrating assay robustness.
