# Supplementary material for: An orthoflavivirus inhibitor targeting multifunctional NS2A protein, a previously unidentified target
Source: PLoS Pathog. 2026 May 5;22(5):e1014190. doi: 10.1371/journal.ppat.1014190 (PMC13166939; doi:10.1371/journal.ppat.1014190)
Supplement: S2 Fig — A Antiviral activity of JNJ-3644 and the seven other isomers against DENV-2/16681 in Vero cells based on GFP expression. B Tetravalent dengue antiviral assay (DENV-1, DENV-2, DENV-3 and DENV-4) of JNJ-3644 in Vero cells using RT-qPCR as readout. C Antiviral activity of JNJ-3644 (green), JNJ-4840 (purple) and JNJ-1953 (orange) in Vero-GFP cells against different orthoflaviviruses (DENV-1, DENV-2, DENV-3, DENV-4, WNV, YFV and ZIKV). D Antiviral activity of JNJ-1953 against ZIKV H/PF/2013 in Huh7 cells based on plaque forming units per mL. (DOCX) [file ppat.1014190.s003.docx]

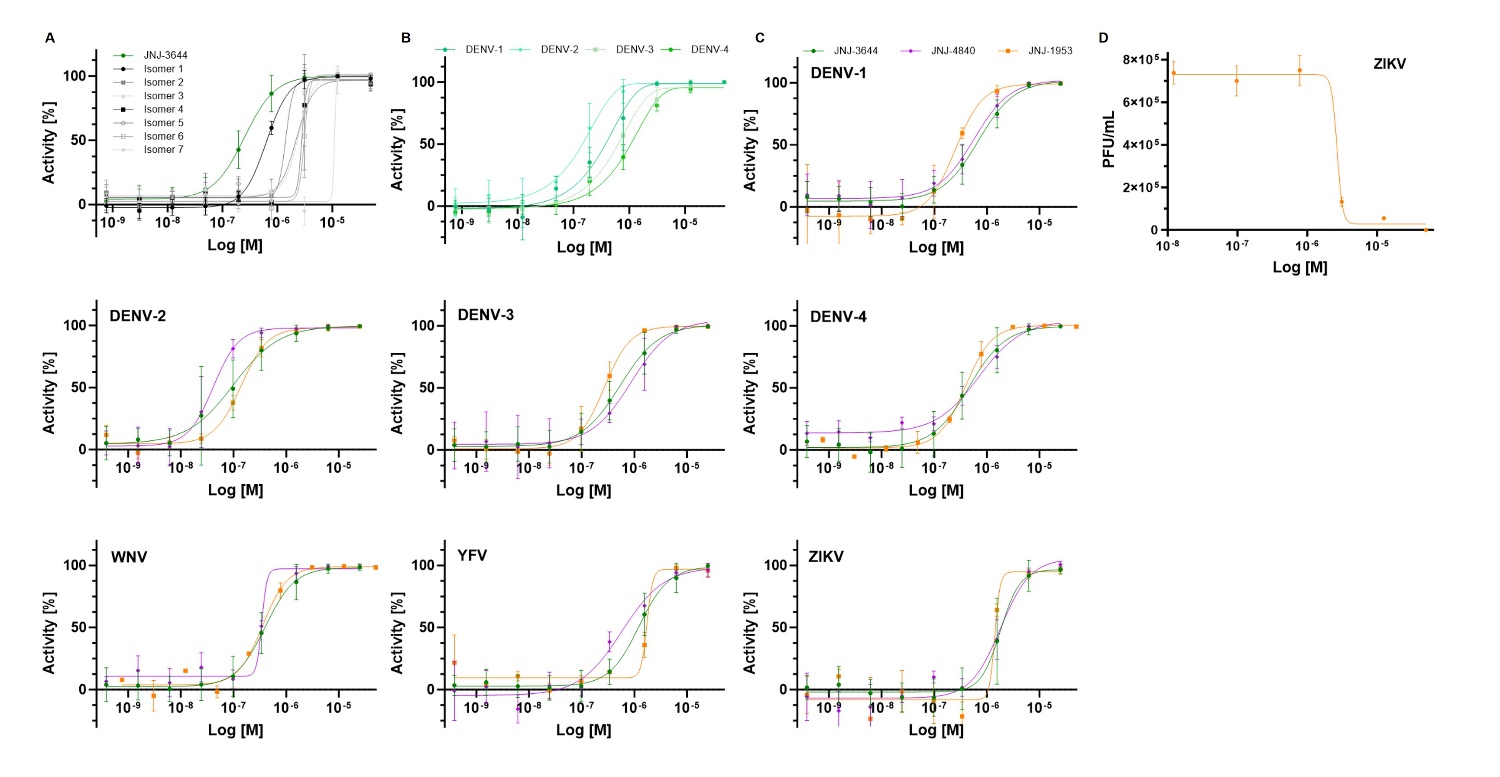


**S2 Fig.: Antiviral activity of JNJ-3644 series in different assays against different orthoflaviviruses. A** Antiviral activity of JNJ‑3644 and the seven other isomers against DENV-2/16681 in Vero cells based on GFP expression. **B** Tetravalent dengue antiviral assay (DENV-1, DENV-2, DENV-3 and DENV-4) of JNJ-3644 in Vero cells using RT-qPCR as readout. **C** Antiviral activity of JNJ-3644 (green), JNJ-4840 (purple) and JNJ-1953 (orange) in Vero-GFP cells against different orthoflaviviruses (DENV-1, DENV-2, DENV-3, DENV-4, WN, YFV and ZIKV). **D** Antiviral activity of JNJ-1953 against ZIKV H/PF/2013 in Huh7 cells based on plaque forming units per mL.
