# Supplementary material for: An orthoflavivirus inhibitor targeting multifunctional NS2A protein, a previously unidentified target
Source: PLoS Pathog. 2026 May 5;22(5):e1014190. doi: 10.1371/journal.ppat.1014190 (PMC13166939; doi:10.1371/journal.ppat.1014190)
Supplement: S3 Fig — A Schematic representation of the subgenomic DENV-2/16681 reporter replicon sgDVs-R2A [69]. B Effect of resistance mutations in NS2A on replication fitness. Resistance mutations identified in Fig 4A were introduced into sgDVs-R2A. Huh7 cells were transfected with 10 µg in vitro transcribed RNA of Wild-type (WT) or mutant sgDVs-R2A and lysed at 48 h post-transfection. Renilla luciferase activity was measured as marker of replication. Relative light units (RLU). Plotted are the mean ± SD from two independent experiments (in duplo), each carried out with independent RNA preparations. (DOCX) [file ppat.1014190.s004.docx]

**
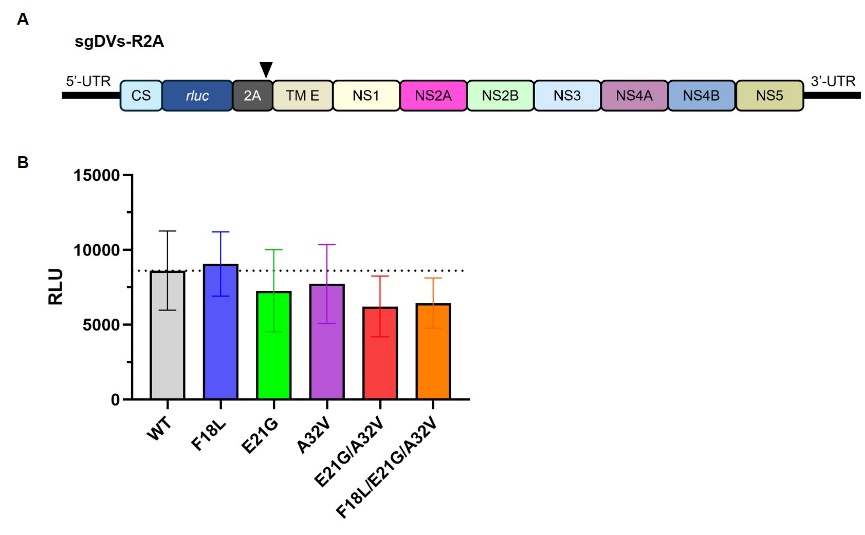
**

**S3 Fig.: Replication properties of resistant subgenomic replicons. A** Schematic representation of the subgenomic DENV-2/16681 reporter replicon sgDVs-R2A (69). **B** Effect of resistance mutations in NS2A protein on replication fitness. Resistance mutations identified in Fig. 4A were introduced into sgDVs-R2A. Huh7 cells were transfected with 10 µg *in vitro* transcribed RNA of Wild-type (WT) or mutant sgDVs-R2A and lysed at 48 h post-transfection. Renilla luciferase activity was measured as marker of replication. Relative light units (RLU). Plotted are the mean ± SD from two independent experiments (in duplo), each carried out with independent RNA preparations.
