# Supplementary material for: An orthoflavivirus inhibitor targeting multifunctional NS2A protein, a previously unidentified target
Source: PLoS Pathog. 2026 May 5;22(5):e1014190. doi: 10.1371/journal.ppat.1014190 (PMC13166939; doi:10.1371/journal.ppat.1014190)
Supplement: S4 Fig — Viral inhibition of the different mutant DENV-2/Eden3295 strains at two concentrations (0.2 and 2 µM) of JNJ-1953. At the highest concentration, JNJ-1953 treatment induces ~40–45% virus reduction for the NS2A mutant viruses, while >99% reduction is observed for the WT DENV-2/EDEN3295 virus strain. (DOCX) [file ppat.1014190.s005.docx]

**
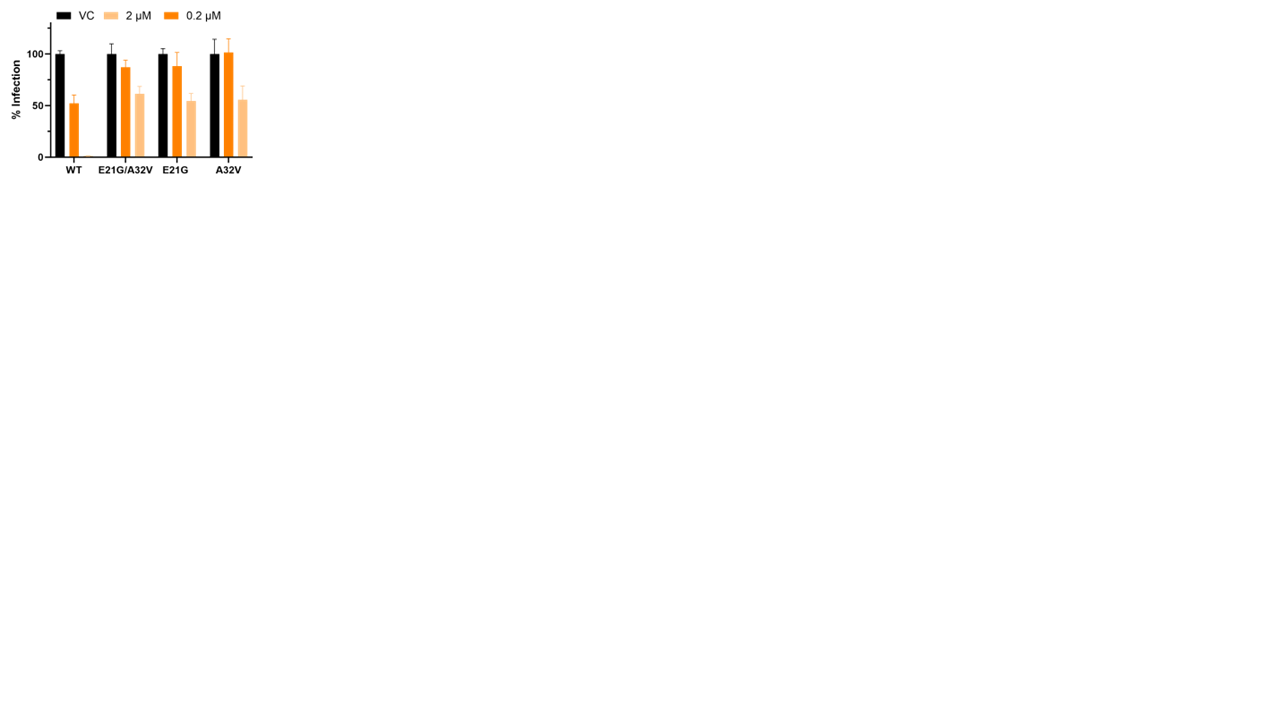
**

**S4 Fig.: Plaque reduction of the different DENV-2/Eden3295 mutant viruses (E21G, A32V and E21G/A32V).** Viral inhibition of the different mutant DENV-2/Eden3295 strains at two concentrations (0.2 and 2 µM) of JNJ‑1953. At the highest concentration, JNJ-1953 treatment induces ~40–45% virus reduction for the NS2A mutant viruses, while >99% reduction is observed for the WT DENV-2/Eden3295 virus strain.
