# Supplementary material for: An orthoflavivirus inhibitor targeting multifunctional NS2A protein, a previously unidentified target
Source: PLoS Pathog. 2026 May 5;22(5):e1014190. doi: 10.1371/journal.ppat.1014190 (PMC13166939; doi:10.1371/journal.ppat.1014190)
Supplement: S5 Fig — A Schematic showing the construct design of DENV-2 NS2A and prM. The constructs design followed Xie et al. [2] B HEK293T cells were transfected with 5 µg each of Myc-prM and Flag-WT, NS2AE21G or NS2AE21G/A32V, followed by treatment with the JNJ-1953, at 6 hours post-transfection. Cell lysates were harvested at 44 hours post-transfection and subjected to co-immunoprecipitation using Myc or IgG control antibody. Western blots showing the detection of Flag-NS2A WT, FLAG-NS2AE21G and Flag-NS2AE21G/A32V immunoprecipitated with Myc-prM in the absence and presence of JNJ-1953. Tubulin was used as a loading control. C Densitometric analysis of the band intensities of WT, NS2AE21G or NS2AE21G/A32V normalized to prM for the co-immunoprecipitated samples upon treatment with JNJ-1953 compared to untreated. Data are presented as bar graphs showing mean with standard deviation from 3 independent experiments. (DOCX) [file ppat.1014190.s006.docx]

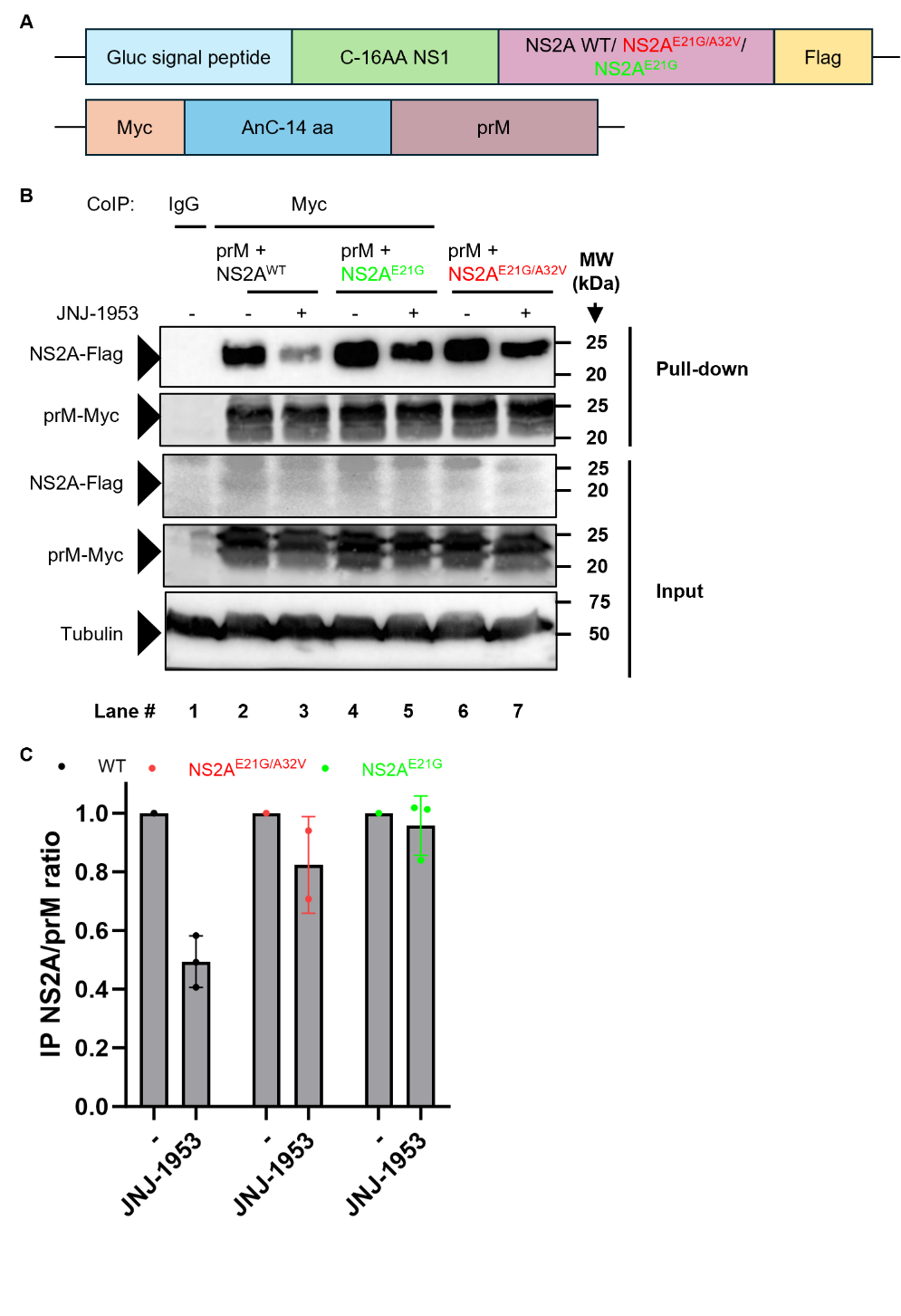


**S5 Fig.: JNJ-1953 destabilizes prM binding to NS2A protein. A** Schematic showing the construct design of DENV-2 NS2A and prM plasmids. The constructs design followed Xie *et al. (2)*  **B** HEK293T cells were transfected with 5 µg each of Myc-prM and Flag-WT, NS2A^E21G^ or NS2A^E21G/A32V^, followed by treatment with the JNJ-1953, at 6 hours post-transfection. Cell lysates were harvested at 44 hours post-transfection and subjected to co-immunoprecipitation using Myc or IgG control antibody. Western blots showing the detection of Flag-NS2A WT, FLAG-NS2A^E21G^ and Flag-NS2A^E21G/A32V^ immunoprecipitated with Myc-prM in the absence and presence of JNJ-1953. Tubulin was used as a loading control. **C** Densitometric analysis of the band intensities of WT, NS2A^E21G^ or NS2A^E21G/A32V^ normalized to prM for the co-immunoprecipitated samples upon treatment with JNJ-1953 compared to untreated. Data are presented as bar graphs showing mean with standard deviation from 3 independent experiments.
