# Supplementary material for: An orthoflavivirus inhibitor targeting multifunctional NS2A protein, a previously unidentified target
Source: PLoS Pathog. 2026 May 5;22(5):e1014190. doi: 10.1371/journal.ppat.1014190 (PMC13166939; doi:10.1371/journal.ppat.1014190)
Supplement: S1 Table — (DOCX) [file ppat.1014190.s007.docx]

**S1 Table: Activity of JNJ-3644 different enantiomers against DENV-2/16681 in Vero cells​**

|  | EC_50_ [µM] | EC_90_ [µM] | CC_50_ [µM] | SI |
| --- | --- | --- | --- | --- |
| JNJ-3644 ​ | 0.12$\pm$0.075 | 0.45$\pm$0.47 | 12$\pm$5.9 | 100 |
| Isomer 1/4497 | 0.63$\pm$ 0.19 | 1.8$\pm1$.1 | 3.7$\pm$0.92 | 6 |
| Isomer 2/4471 | 1.4 $\pm$0.54 | 2.1$\pm$1.2 | 3.1$\pm$2.3 | 2 |
| Isomer 3/3657 | 2.4 $\pm$1.1 | 4.9$\pm$1.3 | 6.4$\pm$0.22 | 3 |
| Isomer 4/4510 | 2.8 $\pm$1.2 | 3.4$\pm$1.2 | 8.6$\pm$3.7 | 3 |
| Isomer 5/4523 | 1.9 $\pm$1.5 | 2.7$\pm$1.9 | 6.6$\pm$2.6 | 3 |
| Isomer 6/3631 | 3.1 $\pm$0.74 | 4.0$\pm$1.0 | 12$\pm$2.7 | 4 |
| Isomer 7/3670 | 11 $\pm$ 4.7 | 12$\pm$5.9 | 25$\pm$3.9 | 2 |

Antiviral data for DENV-2 (Vero) represent mean values from at least 3 independent experiments for the enantiomers. EC_50_ 50% effective concentration. EC_90_ 90% effective concentration. CC_50_ 50% cytotoxic concentration. Selectivity index (SI): ratio CC_50_/EC_50_
