# Supplementary material for: An orthoflavivirus inhibitor targeting multifunctional NS2A protein, a previously unidentified target
Source: PLoS Pathog. 2026 May 5;22(5):e1014190. doi: 10.1371/journal.ppat.1014190 (PMC13166939; doi:10.1371/journal.ppat.1014190)
Supplement: S2 Table — (DOCX) [file ppat.1014190.s008.docx]

S2 Table: Antiviral activity of JNJ-3644 against other RNA and DNA viruses

| Cells | Virus | EC_50_ [µM] | CC_50_ [µM] | SI |
| --- | --- | --- | --- | --- |
| HeLa | RSV | 16 $\pm$ 2.8 | 20 $\pm0.6$ | 1 |
| Huh7 | CHIKV | >50 | 8.4 |  |
| Huh7-Luc | HCV | 7.9 $\pm$ 0.74 | 19 $\pm$ 0.09 | 2 |
| LLC-MK2 | hMPV | 6.9 $\pm$ 4.4 | 9.7 $\pm$ 1.5 | 1 |
| A549 | FLU – SG | 2.1 $\pm$ 1.1 | 4.6$\pm$ 1.9 | 2 |
| A549 | FLU – TW | 3.1 $\pm$ 1.4 | 4.6$\pm$ 1.9 | 1 |
| HepG2.117 | HBV | >50 | 20 $\pm$ 2.7 |  |

Antiviral data represents mean values from two independently performed experiments. EC_50_ 50% effective concentration. CC_50_ 50% cytotoxic concentration. Selectivity index (SI): ratio CC_50_/EC_50_. RSV: respiratory syncytial virus. CHIKV: Chikungunya virus. HCV: Hepatitis C Virus. hMPV: human metapneumovirus. Flu: Influenza virus. SG: Singapore. TW: Taiwan. HBV: Hepatitis B virus.
