# Supplementary material for: An orthoflavivirus inhibitor targeting multifunctional NS2A protein, a previously unidentified target
Source: PLoS Pathog. 2026 May 5;22(5):e1014190. doi: 10.1371/journal.ppat.1014190 (PMC13166939; doi:10.1371/journal.ppat.1014190)
Supplement: S3 Table — (DOCX) [file ppat.1014190.s009.docx]

S3 Table: Percentage of allele frequency of the mutations developed in the DENV-2/16681 strain at passage 13 following an *in vitro* resistance experiment using JNJ-3644

| **Mutation in DENV-2/16681** | **Allele frequency (%)** | |
| --- | --- | --- |
|  | Passage 13 | Control |
| 2K_Q3S | 0.28 | 0.01 |
| 2K_D4N | 0.30 | 0.01 |
| 2K_N5K | 0.29 | 0.01 |
| C_N93D | 0.21 | 0.02 |
| NS1_L338M | 0.20 | 0.01 |
| NS2A_F18L | 0.20 | 0.04 |
| NS2A_E21G | 0.43 | 0.01 |
| NS2A_A32V | 0.53 | 0.02 |
| NS2A_L211P | 0.21 | 0.01 |
| NS2B_E92D | 0.27 | 0.03 |
| NS3_L216S | 0.24 | 0.01 |
| NS3_R217N | 0.25 | 0.01 |
| NS3_E255V | 0.22 | 0.01 |
| NS5_W703Q | 0.22 | 0.02 |
| NS5_T751R | 0.24 | 0.01 |

Drug-resistant variants were selected by passaging DENV-2/16681 in the presence of gradually increasing concentrations of JNJ-3644. CPE was present at passage 13 in cells treated with 5 µM JNJ-3644. Next-generation sequencing (NGS) was performed on DENV-2 variants harvested at the end of the experiment (p13). Fifteen individual mutations were identified at the end point, which were not present in the in-parallel-passaged untreated cultures. The mutations in NS2A: F18L, E21G, and A32V (shown in bold) were present in all three In vitro resistance selection (IVRS) experiments compared to the other mutations (S4 Table and S5 Table).
