# Supplementary material for: An orthoflavivirus inhibitor targeting multifunctional NS2A protein, a previously unidentified target
Source: PLoS Pathog. 2026 May 5;22(5):e1014190. doi: 10.1371/journal.ppat.1014190 (PMC13166939; doi:10.1371/journal.ppat.1014190)
Supplement: S4 Table — (DOCX) [file ppat.1014190.s010.docx]

S4 Table: Percentage of allele frequency of the mutations developed in the DENV-2/16681 strain at passage 18 following an *in vitro* resistance experiment using JNJ-1953

| **Mutation in DENV-2/16681** | **Allele frequency (%)** | |
| --- | --- | --- |
|  | Passage 13 | Control |
| NS1_A43V | 0.27 | 0.01 |
| NS1_K227E | 0.25 | 0.01 |
| NS1_H261Q | 0.64 | 0.06 |
| NS2A_F18L | 0.20 | 0.02 |
| NS2A_E21G | 0.46 | 0.01 |
| NS2A_A32V | 0.27 | 0.01 |
| NS2A_L181F | 0.99 | 0.04 |
| NS2A_R213N | 0.32 | 0.06 |
| NS4A_M85V | 0.25 | 0.01 |
| NS5_E733Q | 0.20 | 0.07 |

Drug-resistant variants were selected by passaging DENV-2/16681 in the presence of gradually increasing concentrations of JNJ-1953. CPE was present at passage 18 in cells treated with 5 µM JNJ-3644. NGS was performed on DENV-2 variants harvested at the end of the experiment (p18). Ten individual mutations were identified at the end point, which were not present in the in-parallel-passaged untreated cultures. The mutations in NS2A: F18L, E21G, and A32V (shown in bold) were present in all three IVRS experiments compared to the other mutations (S3 Table and S5 Table).
