# Supplementary material for: An orthoflavivirus inhibitor targeting multifunctional NS2A protein, a previously unidentified target
Source: PLoS Pathog. 2026 May 5;22(5):e1014190. doi: 10.1371/journal.ppat.1014190 (PMC13166939; doi:10.1371/journal.ppat.1014190)
Supplement: S5 Table — (DOCX) [file ppat.1014190.s011.docx]

S5 Table: Percentage of allele frequency of the mutations developed in the DENV-2/16681 strain at passage 18 and passage 28 following an *in vitro* resistance experiment using JNJ-1953

| **Mutation in DENV-2/16681** | **Allele frequency (%)** | | |
| --- | --- | --- | --- |
|  | Passage 18 | Passage 28 | Control |
| E_K122E/I | 0.54 | 0.25 | 0.05 |
| E_T454I | 0.37 | 0.21 | 0.01 |
| M_V99A | 0.21 | 0.24 | 0.01 |
| NS1_G235E | 0.23 | 0.23 | 0.01 |
| NS2A_F18L | 0.52 | 0.32 | 0.01 |
| NS2A_E21G | 0.34 | 0.28 | 0.00 |
| NS2A_A32V | 0.14 | 0.40 | 0.00 |
| NS4A_E9G | 0 | 0.55 | 0.00 |
| NS4B_T179I | 0.26 | 0.84 | 0.01 |
| NS5_K46R | 0.03 | 0.56 | 0.01 |

Drug-resistant variants were selected by passaging DENV-2/16681 in the presence of gradually increasing concentrations of JNJ-1953. CPE was observed starting at passage 18 and completely presented at passage 28 in cells treated with 2.5 µM JNJ-1953. Following JNJ-1953 exposure, passage 18 and 28 viruses are subsequently characterized using NGS. Eleven individual mutations were identified at the end point, which were not present in the in-parallel-passaged untreated cultures. The mutations in NS2A: F18L, E21G, and A32V (shown in bold) were present in all three IVRS experiments compared to the other mutations (S3 Table and S4 Table).
