# Supplementary material for: An orthoflavivirus inhibitor targeting multifunctional NS2A protein, a previously unidentified target
Source: PLoS Pathog. 2026 May 5;22(5):e1014190. doi: 10.1371/journal.ppat.1014190 (PMC13166939; doi:10.1371/journal.ppat.1014190)
Supplement: S6 Table — (DOCX) [file ppat.1014190.s012.docx]

S6 Table: Natural occurrence of the NS2A mutation residues in clinical isolates

| **Compares to** **DENV-2/16681** (*) | **Orthoflavivirus frequency mutant in clinical isolates (%)** | | | | | | | | |
| --- | --- | --- | --- | --- | --- | --- | --- | --- | --- |
|  | DENV-2 | DENV-1 | DENV-3 | DENV-4 | JEV | WNV | YFV | ZIKV |  |
| F18L | 0.65% | 1.4% | 100% | 0.1% | 0% | 0% | 0% | 0% |  |
| E21G | 0% | 0% | 0% | 0% | 0% | 0% | 0% | 0% |  |
| A32V | 0.2% | 1.6% | 0% | 0% | 0% | 0% | 0% | 0% |  |

The natural occurrence of the NS2A mutations in clinical isolates was retrieved from the Bacterial and Viral bioinformatics Research center (BV-BRC) database (retrieved in 2024). Prevalence values of ≤0.1% are not shown.

* Kinney RM, Butrapet S, Chang GJ, Tsuchiya KR, Roehrig JT, Bhamarapravati N, et al. Construction of infectious cDNA clones for dengue 2 virus: strain 16681 and its attenuated vaccine derivative, strain PDK-53. Virology. 1997;230(2):300-8.
