# Supplementary material for: An orthoflavivirus inhibitor targeting multifunctional NS2A protein, a previously unidentified target
Source: PLoS Pathog. 2026 May 5;22(5):e1014190. doi: 10.1371/journal.ppat.1014190 (PMC13166939; doi:10.1371/journal.ppat.1014190)
Supplement: S7 Table — (DOCX) [file ppat.1014190.s013.docx]

S7 Table: Antiviral activity of JNJ-3644 and JNJ-4840 against WT and NS2A mutant DENV-2 subgenomic constructs

| **pFK-sgDVs-R2A** | **JNJ-3644** | | **JNJ-4840** | |
| --- | --- | --- | --- | --- |
| Cells | EC_50_ [µM] | FC | EC_50_ [µM] | FC |
| WT | 0.98 |  | 0.23 |  |
| E21G | 5.3 | 5 | 5.4 | 24 |
| A32V | 3.4 | 3 | 3.1 | 14 |
| F18L | 3.9 | 4 | 3.8 | 17 |
| E21G/A32V | 4.2 | 4 | 5.1 | 23 |
| F18L/E21G/A32V | 4.4 | 5 | 5.8 | 26 |

Antiviral data represents mean values from two independently performed experiments. EC_50_ 50% effective concentration. FC: Fold change compared to WT.
